# *WaaL–WaaP* receptor cross-talk drives phage-induced collateral sensitivity to colistin in *Salmonella* Pullorum

**DOI:** 10.1186/s13567-026-01825-8

**Published:** 2026-08-12

**Authors:** Mianzhi Wang, Yiwen Ren, Lei Jiang, Zixuan Wang, Peng Liu, Mingyi Zhang, Junxuan Zhang, Ruichao Li, Yongxue Sun, Zhiqiang Wang

**Affiliations:** 1 College of Veterinary Medicine, Yangzhou University, Yangzhou, 225009 Jiangsu China; 2 Jiangsu Co-Innovation Center for Prevention and Control of Important Animal Infectious Diseases and Zoonoses, Yangzhou, 225009 China; 3 Jiangsu Interdisciplinary Center for Zoonoses and Biosafety, Yangzhou University, Yangzhou, 225009 China; 4 College of Veterinary Medicine, South China Agricultural University, Guangzhou, 510642 China; 5 National Risk Assessment Laboratory for Antimicrobial Resistance of Animal Original Bacteria, Guangzhou, 510642 China

**Keywords:** *Salmonella* Pullorum, phage, colistin, LPS, fitness cost

## Abstract

Phage therapy typically fails when bacteria rapidly evolve resistance, yet the inevitable fitness costs of escape mutants remain underexploited. In this study, an iterative and targeted phage-cocktail formulation strategy (ITPFS) was presented and developed. A potent lytic phage, PHZ055, was firstly isolated against a colistin-resistant *Salmonella* Pullorum strain. Co-cultured until resistant mutants emerged, a second lytic phage, PYW047_3, was screened for the resistant variant. The two phages were then combined as a cocktail. Results showed that bacterial growth was strongly suppressed and the emergence of resistance was markedly delayed. Receptor identification revealed that both phages use lipopolysaccharide (LPS) as a receptor, but at distinct sites: PHZ055 targets *WaaL*, and PYW047_3 targets *WaaP*. Mutation of *WaaL* was recently shown to be responsible for the loss of polymerized O-antigen units and thereby exposed the PYW047_3 binding region, indicating that phage cocktails targeting distinct receptor sites can be directionally screened. Moreover, mutants that escaped the two phages not only incurred a growth cost but also exhibited a 32-fold increase in colistin sensitivity. Combining the two phages together with 1/4 MIC colistin almost eradicated all bacteria within 24 h. In all, our study successfully provides a repeatable strategy that incorporates adaptive trade-offs to boost therapeutic efficacy.

**Supplementary Information:**

The online version contains supplementary material available at 10.1186/s13567-026-01825-8.

## Introduction

*Salmonella enterica* subsp. *enterica* serovar Pullorum (*S.* Pullorum) is a pathogen that exclusively infects poultry, causing acute septicemia in chicks and chronic asymptomatic infections in adult birds, along with impairment of the reproductive system [1–3]. It can induce extremely high mortality, potentially close to 100%, in young chickens and turkeys during the first 2–3 weeks post-hatch [4], and the disease is particularly prevalent in Asia, where it causes substantial economic losses [5]. Nowadays, the rise of antimicrobial resistance (AMR) has been recognized as an emerging threat to public health [6], and phages are considered an effective weapon against these drug-resistant strains [7], including *S*. Pullorum [8]. However, the rapid development of phage resistance still limits their therapeutic efficacy [9].

Although the emergence of phage-resistant bacteria is inevitable, there is growing evidence showing that phage-imposed selection drives mutations, enabling bacteria to evade infection but with a fitness cost [10]. There is a growing body of literature showing that the fitness trade-off in phage-resistant bacteria includes growth defects [11], inability to form biofilm [12, 13], attenuated virulence [14–16], enhanced clearance by immune cells [17], host colonization defects [18], and increased sensitivity to antibiotics [17, 19-23]. Therefore, strategically exploiting the fitness costs inherent to phage resistance represents a promising approach for improving the clinical therapeutic success of phage therapy.

Typically, for successful phage therapy, the phage must complete its entire lytic cycle of receptor adsorption, genome injection, virion assembly, and release [24]. In many instances, lytic phages must first recognize and adsorb to receptors on the bacterial surface [25]. Given that evolved phage resistance often involves mutations in bacterial cell surface receptors that are important for cellular stability and function [26], and '“steering” survivors towards deleterious surface mutations can potentially improve treatment outcomes [27], it is clear that taking advantage of these receptor changes to create a combinatorial phage cocktail will be the first important step in enhancing the cocktail’s efficacy.

However, we still lack robust strategies to systematically capture and exploit the synergistic potential embedded in surface mutations because the evolutionary trajectory of resistance mutations induced by different phage combinations is unpredictable, thus probably failing in phage therapy [23, 28]. Consequently, several studies have innovatively proposed that phage cocktails targeting distinct receptors can delay the emergence of phage-resistant mutants and enhance the overall efficacy of phage combinations [29–31]. It is noteworthy that, although dual-receptor phage cocktails have delayed the emergence of phage-resistant bacteria, the cocktail is merely the arithmetic sum of the two phages acting alone. On the contrary, a further iterative and targeted phage cocktail strategy is likely to be more valuable [32, 33] when targeting an isolated second-generation lytic phage against phage-resistant bacterial mutants under pressure from the first-generation phage, as this cocktail effectively exploits the fitness costs imposed by receptor modification.

Importantly, the fitness tradeoff of collateral sensitivity to antibiotics can also be exploited and combined with the iterative phage cocktail, as this phenomenon has been widely observed in diverse pathogens, including imipenem, meropenem, colistin, tigecycline, and ciprofloxacin in *Klebsiella pneumoniae* [19, 22, 23], colistin, erythromycin, doxycycline, gentamicin, ceftiofur, meropenem, and sulfadiazine in *Salmonella enterica* serovar Enteritidis [20], beta-lactam antibiotics in *Acinetobacter baumannii* [21], and several outer membrane-targeting antibiotics in *Escherichia coli* [17]. It is not surprising that phage-driven surface modification can simultaneously confer collateral sensitivity to antibiotics because these compounds directly kill the bacteria by acting on the cell wall or membrane [34]. Therefore, combination therapies with an iterative and targeted phage cocktail and re-sensitized antibiotics are clinically promising, and it is also different from the traditional phage–antibiotic synergy (PAS) [35] and can be proposed as an innovative therapeutic option for antimicrobial-resistant bacteria.

In this study, we develop a comprehensive iterative and targeted phage cocktail formulation strategy against pathogenic *S*. Pullorum strains HZ055 collected from a chicken farm with high mortality for embryos. The approach was designed to achieve two synergistic objectives: (i) to assemble a combinatorial phage cocktail capable of iteratively suppressing emergent resistance strains in receptor modifications (*WaaL* and *WaaP* cross-talk in a lipopolysaccharide (LPS) receptor in this study), and (ii) to exploit the evolutionary trade-offs in collateral sensitivity to antibiotics (colistin in this study). This framework demonstrated the cocktail’s extremely high efficacy in the treatment of *S.* Pullorum strains HZ055, showing the clinical potential of our strategy.

## Materials and methods

### Isolation and identification of strains HZ055 and YW047

Dead chicken embryos were collected from a chicken farm in Jiangsu Province and cultured in Rappaport Vassiliadis (RV) Salmonella enrichment broth. The overnight medium was then selectively cultured in xylose lysine desoxycholate (XLD) broth at 37 °C for 12 h. The single colony was subsequently recovered and identified in Salmonella Shigella (SS) agar medium. The following day, single colonies were selected for sequencing to complete the isolation and identification. Finally, two multidrug-resistant (MDR) pathogenic *S.* Pullorum strains, HZ055 and YW047, were selected as host bacteria for the later experiments; they are detailed in Additional file 1.

### Isolation and identification of lytic phages PHZ055 and PYW047_3

Phages PHZ055 and PYW047_3 were isolated from municipal chicken farm wastewater with *S.* Pullorum ATCC9120 serving as the host, according to our previous study [36]. Briefly, raw sewage was pre-filtered through a 0.22-µm Millex-GP membrane (Millipore, Burlington, MA, USA). The clarified filtrate was mixed with an equal volume of log-phase ATCC9120 culture and incubated overnight at 37 °C with shaking at 200 rpm. Lysis was confirmed by spot tests. Single discrete plaques were picked and purified through three consecutive rounds of double-layer agar plating. High-titer stocks were then stored in SM buffer (100 mM NaCl, 8 mM MgSO₄, 50 mM Tris–HCl, pH 7.5) and stored at 4 °C; they are detailed in Additional file 1.

### Phage morphological observation and characteristics

Phages PHZ055 and PYW047_3 were applied to a carbon-coated formvar grid, allowed to adsorb for 15 min, negatively stained with 2% (w/v) phosphotungstic acid (pH 7.0), and air-dried. Samples were examined in an FEI Tecnai transmission electron microscope (Thermo Fisher, Hillsboro, OR, USA) operated at 80 kV to conduct morphological observation.

A phage suspension (10⁸ PFU/mL, 5 mL) was exposed to UV light in a biosafety cabinet for 0, 20, 40, 60, 80, 100, or 120 min to detect ultraviolet (UV) stability. Another phage suspension was held for 1 h at −80 °C, −20 °C, 4 °C, 37 °C, 50 °C or 60 °C to detect thermal stability. LB medium was adjusted to pH 1, 3, 5, 7, 9, or 11 with HCl or NaOH at a pre-warmed temperature of 37 °C and incubated at 37 °C for 60 min to detect pH tolerance.

Optimal multiplicity of infection (MOI) was determined by infecting early-log-phase cultures (about 10⁷ colony-forming units [CFU]/mL) with phage at MOI values of 100, 10, 1, 0.1, 0.01, and 0.001. After 5 h incubation at 37 °C with shaking (200 rpm), cultures were chloroform-treated and titrated in duplicate by the double-layer agar method; the assay was performed in triplicate. PFU/mL values were calculated from spot counts.

A one-step growth curve was also detected. Phages were mixed with an exponential-phase culture at an optimal MOI and allowed to adsorb for 10 min at room temperature. The mixture was then centrifuged at 2700 × *g* for 10 min to remove unabsorbed virions, and the infected cells were resuspended in 5 mL of fresh LB medium. Every 10 min, 200 µL of the culture was sampled, immediately clarified by centrifugation (10,000 × *g*, 2 min), and filtered through a 0.22-µm membrane. Then 100 µL of the filtrate was serially diluted for phage titer determination. Phage titers were determined in duplicate by the double-layer agar method; the experiment was performed in triplicate.

### Isolation and confirmation of phage-resistant mutants

Phage PHZ055-resistant mutants (055R) were isolated and confirmed as previously described [37–39] with minor modifications. Briefly, *S.* Pullorum strain HZ055 (derived from a single ancestral colony) and phage PHZ055 were co-cultured in 2 mL LB broth at a starting bacterial concentration of approximately 1 × 10⁶ CFU/mL and optimal phage MOI. At 12-h intervals, 200 µL aliquots of the co-culture were collected and centrifuged at 2700 × *g*. The resulting cell pellets were resuspended in fresh LB broth, and phage PHZ055 was supplemented to optimal MOI to maintain continuous selective pressure before initiating the next incubation cycle. This subculture process was repeated for six transfers over a 3-day period. Following the final incubation interval, bacterial cells were again pelleted by centrifugation, resuspended in fresh LB broth, and spread-plated onto LB agar plates to recover surviving bacterial clones. Phage PHZ055-resistant mutants were determined using spot assays against the PHZ055 phage; 055R were thereby isolated and confirmed. Additionally, phage PHZ047_3-resistant mutants (047R) and dual-resistant mutants against both phage PHZ055 and phage PHZ047_3 (R2P) were also isolated and confirmed following the same protocol described above.

### Phage DNA extraction, genome sequencing, assembly, and annotation

Purified phage lysates were used for extraction of genome DNA as described previously [40, 41]. Shortly, lysates were precipitated with 10% (w/v) PEG-8000, pelleted, and resuspended in SM buffer. Residual free DNA was removed by DNase I treatment (100 U/mL; Sangon Biotech, Shanghai, China). Phage DNA was extracted by phenol–chloroform–isoamyl alcohol extraction and ethanol precipitation. The final DNA pellet was dissolved in TE buffer (10 mM Tris–HCl, 1 mM EDTA, pH 8.0) and quantified with a NanoDrop spectrophotometer (Thermo Fisher, Wilmington, DE, USA).

Genomic DNA of PHZ055 and PYW047_3, including the mutants 055R, 047R, and R2P, was sequenced on an Illumina HiSeq 2500 platform (2 × 150-bp paired-end reads; Genewiz, Suzhou, China). Long reads were also generated on an Oxford Nanopore MinION using R9.4.1 flow cells and the RBK004 barcoding kit, following the manufacturer's instructions and previously published protocols [42]. Illumina reads were quality-trimmed with Trimmomatic and assembled with SPAdes v3.15; contigs < 500 bp were discarded [43]. Nanopore reads were base-called with Guppy and assembled de novo with Flye v2.9. Hybrid assembly of both datasets was performed with Unicycler to generate complete, circular genomes.

Bioinformatics analyses were performed as follows: Antimicrobial resistance (AMR) genes and virulence genes identified using ABRicate v1.0.1 with the ResFinder databases, respectively. The distribution of AMR genes and virulence genes were visualized using GraphPad Prism 9. Functional annotation of complete genomes was performed via submission to the RAST (Rapid Annotation using Subsystem Technology) online platform. Open reading frames (ORFs) were predicted with Prodigal and annotated automatically. The start coding sequence (CDS) for each predicted ORF was determined based on the presence of a canonical start codon (ATG, GTG, or TTG), a ribosome-binding site (Shine-Dalgarno sequence) within an appropriate distance (4–10 bp upstream), and a minimum coding length of 150 bp. Whole-genome maps were generated with CGView Server. Phage genera was confirmed according to the International Committee on Taxonomy of Viruses (ICTV) website. The evolutionary relationships were analyzed using phylogenetic trees based on the whole genome against the National Center for Biotechnology Information (NCBI) representative RefSeq database. The official database link involved in our research can be found in Additional file 2.

### Whole-genome sequencing, mutation identification, and construction of gene knockout strains

Genomic DNA of the parental strains HZ055 and YW047 and the phage-resistant mutants was extracted using the FastPure Bacteria DNA Isolation Mini Kit (Vazyme, Nanjing, China). After quality assessment using a microspectrophotometer, qualified DNA samples were sent to Sangon Biotech (Shanghai, China) for library construction and sequencing. Sequencing was performed on the Illumina NovaSeq 6000 platform using a paired-end 150-bp (PE150) strategy. Genome assembly was performed using SPAdes v3.15, and contigs shorter than 500 bp were discarded. The assembled genome sequences were submitted to the RAST online platform for gene prediction and functional annotation. To identify mutations in the resistant mutants, sequencing reads of each resistant mutant were aligned to the respective reference genomes (HZ055 and YW047) using BWA software. Single nucleotide polymorphisms (SNPs) and insertions/deletions (InDels) were detected and filtered using GATK with the following criteria: sequencing depth ≥ 10 × , quality value ≥ 30, and variant frequency ≥ 90% in the mutant. Functional annotation of the detected variants was performed using SnpEff combined with the RAST annotation results to determine the gene region, mutation type, and potential amino acid changes, with a focus on genes potentially involved in phage receptor functions. Key mutations identified by whole-genome sequencing were further validated by Sanger sequencing.

To generate knockout mutants of potential phage receptor genes in HZ055 and YW047, target genes were selected based on prior SNP analysis using gene-specific primers (Additional file 3): *yehA* (fimbria synthesis) and *waaL* (LPS synthesis) in HZ055; *waaP* and *rfaJ* (both LPS synthesis) in YW047. About 600-bp flanking homology arms for each target were designed, and primers with 5′ Xma I/Xba I sites were synthesized. Overlap extension polymerase chain reaction (PCR) with wild-type genomic DNA as templates amplified HZ055Δ*yehA*, HZ055Δ*waaL*, YW047Δ*waaP*, and YW047Δ*rfaJ* fragments. These fragments and suicide plasmid pRE112 were digested with Xma I/Xba I then directionally ligated to construct recombinant plasmids (pRE112-Δgene).

Electrocompetent *E. coli* χ7213 (prepared by 10% glycerol washing) were electroporated with pRE112-Δgene plasmids. Transformants were selected on LB agar with 50 μg/mL DAP and 24 μg/mL chloramphenicol (Cm) used as donor strains and verified by PCR. Donor and recipient strains HZ055/YW047 were mixed in a 1:1 ratio, spotted on LB agar with 50 μg/mL DAP, and statically incubated at 37 °C for 8 h for conjugation. Conjugants were serially diluted and plated on Salmonella-selective XLD agar with 25 μg/mL Cm, then incubated at 37 °C for 36 h to select Cm-resistant (Cm) single-crossover integrants (first homologous recombination).

Ten single-crossover colonies were cultured in LB with Cm (37 °C, 8 h shaking) to induce second homologous recombination. Cultures were serially diluted and plated on LB agar with 15% sucrose (30 °C, overnight). Sucrose-resistant/Cm-sensitive clones were selected as candidate knockout mutants. PCR verification with primers about 600 bp upstream/downstream of targets showed about 1.2-kb amplicons for mutants (target deletion) versus larger fragments for wild types.

### Gene complementation

To restore the function of *waaL* and *waaP*, the full-length *waaL* gene from wild-type (WT) HZ055 and *waaP* gene from WT YW047 were cloned into their respective knockout mutant backgrounds (HZ055Δ*waaL* and YW047Δ*waaP*). Electrocompetent cells of HZ055Δ*waaL* and YW047Δ*waaP* were prepared and stored at −80 °C until use. The pBAD44 vector was used for complementation experiments. Homologous recombination primers (WaaL-3F/R and WaaP-3F/R) for amplifying *waaL* and *waaP*, respectively, and verification primers for pBAD44 (pBAD44-F/R) were designed using the Vazyme primer design platform. The *waaL*-3 and *waaP*-3 fragments were PCR-amplified from genomic DNA of HZ055 WT and YW047 WT, respectively.

These amplicons were assembled with EcoRI/HindIII-digested linearized pBAD44 using the Vazyme ClonExpress II One Step Cloning Kit to generate recombinant plasmids pBAD44-*waaL* and pBAD44-*waaP*. The recombinant plasmids were heat-shocked into *E. coli* DH5α competent cells for propagation. Transformants were selected on kanamycin-supplemented agar plates, and positive clones were verified by colony PCR using pBAD44-specific verification primers. Plasmids were extracted from verified clones and subsequently electroporated into electrocompetent HZ055Δ*waaL* and YW047Δ*waaP* cells to generate complemented strains.

### Efficiency of plating (EOP) assay

The EOP assay was performed to quantify the lytic efficiency of diverse phages from our phage bank against bacterial strains. Briefly, tenfold serial dilutions of phage (10^–1^ to 10⁻⁶ PFU/mL) were prepared in sterile PBS. A 5-μL aliquot of each dilution was spotted onto soft LB agar plates (0.7% agar) pre-mixed with 100 μL logarithmic-phase bacterial culture (10⁸ CFU/mL). Plates were incubated overnight at 37 °C, and plaque numbers were counted. The EOP was calculated as the ratio of plaque-forming units (PFU) on the test strain to (EOP = PFU_test_/PFU_wild-type_). All assays were performed in triplicate, and results are presented as the mean ± standard deviation (SD).

### Comparative phage adsorption efficiency assay

To compare the adsorption efficiency of phages to different bacterial strains, a single time-point adsorption experiment was performed. Logarithmic-phase bacterial cultures were mixed with the optimal phage MOI, followed by incubation at 37 °C for 8 min. The mixture was centrifuged at 3000 × *g* for 5 min, and the supernatant was filtered through a 0.22-μm membrane to remove residual bacterial cells. Phage titers in the filtrate were determined via the double-layer agar method, and the adsorption efficiency was calculated as [(initial phage titer) − (unadsorbed phage titer)]/(initial phage titer) × 100%. All assays were performed in triplicate.

### Antibiotic susceptibility assay

Minimum inhibitory concentrations (MICs) of 13 antimicrobials (ampicillin, meropenem, ticarcillin, tigecycline, kanamycin, tobramycin, colistin, norfloxacin, streptomycin, rifampin, doxycycline, ceftazidime, and vancomycin) were determined by the standard broth microdilution method, according to the Clinical and Laboratory Standards Institute (CLSI) guideline [44]. Briefly, eight disinfectants were serially diluted twofold in Mueller–Hinton broth (MHB) and mixed with an equal volume of bacterial suspension (≈1.5 × 10⁶ CFU/mL) in UV-sterilized, clear 96-well microtiter plates (Corning, NY, USA). After 18 h at 37 °C, the MIC was recorded as the lowest concentration showing no visible growth. All assays were performed in triplicate.

### Time-kill kinetics of phage or phage–antibiotic combination

The bactericidal activity of phage alone or combined with antibiotic was evaluated against strain HZ055. The experiments were randomly divided into five groups: (1) PBS-negative group, (2) PHZ055 monotherapy group, (3) PHZ055 + PYW047_3 combination therapy group, (4) PHZ055 + PYW047_3 + 1/4 MIC colistin (2P + 1/4mic) therapy group, and (5) PHZ055 + PYW047_3 + 1/8 MIC colistin (2P + 1/8mic) therapy group. The microplate was incubated at 37 °C with orbital shaking at 180 rpm, and OD₆₀₀ values were recorded every 3 h for 24 h using a Synergy H1 microplate reader (BioTek Instruments, Winooski, VT, USA). Each strain was tested in three technical replicates, and the experiment was independently repeated three times.

### Virulence assessment in the *Galleria mellonella* model

The virulence of HZ055 and its derivative strains (055R, HZ055Δ*YehA*, HZ055Δ*WaaL*, and R2P) was evaluated using the *G. mellonella* infection model. *Galleria mellonella* with a body weight of 250–300 mg was randomly divided into seven groups (12 per group). The groups were as follows: (1) PBS-negative group, (2) HZ055 infection group, (3) YW047 infection group, (4) HZ055Δ*WaaL* infection group, (5) YW047Δ*WaaP* infection group, (6) 055R infection group, and (7) R2P infection group. The experiment was repeated three times independently.

Bacterial suspensions of different concentrations (10 μL per *G. mellonella*) were injected into the penultimate proleg of each larva using a microinjector. Based on the preliminary virulence characterization of HZ055, the infectious dose was standardized to the 75% lethal dose (1 × 10⁷ colony-forming units [CFU]/mL). Infected *G. mellonella* were incubated at 37 °C in the dark and monitored continuously for 36 h to assess strain virulence. Larvae were considered dead if they showed no response to physical stimulation.

### Scanning electron microscopy (SEM) observation

HZ055, 055R, HZ055Δ*WaaL*, and R2P strains were cultured to the mid-logarithmic growth phase, harvested by centrifugation, and washed three times with PBS buffer (pH 7.4). Bacterial pellets were fixed overnight at 4 °C in 2.5% glutaraldehyde solution. Fixed samples were dehydrated through a graded ethanol series (30%, 50%, 70%, 80%, 90%, 95%, and 100%), followed by critical-point drying and sputter-coating with platinum. Processed samples were mounted on SEM sample stubs and observed using a field-emission scanning electron microscope (ZEISS Sigma 300, Carl Zeiss AG, Oberkochen, Germany) at the Zhongke Baice Technology Platform. High-resolution images of bacterial surface morphology were acquired by scanning the sample surface with a focused electron beam.

### Assessment of bacterial membrane fluidity and permeability

HZ055, 055R, HZ055Δ*WaaL*, YW047, YW047Δ*WaaP*, and R2P strains were cultured to the mid-logarithmic growth phase, resuspended in PBS buffer, and adjusted to an OD₆₀₀ of 0.3. Fluorescent probes were added to the bacterial suspensions as follows: Disc3(5) for membrane potential, Laurdan for membrane fluidity, and propidium iodide (PI) and *N*-phenyl-1-naphthylamine (NPN) for membrane permeability and outer membrane permeability, respectively. All samples were incubated in the dark at 37 °C with shaking at 180 rpm for 30 min. After incubation, 200 μL of each bacterial suspension was transferred to a black 96-well microplate, with three replicate wells per treatment. A negative control group (without fluorescent probes) was included in parallel.

For membrane fluidity analysis, fluorescence intensity was measured using a fluorescence spectrophotometer (Thermo Fisher Scientific, Waltham, MA, USA) with an excitation wavelength of 350 nm, and emission wavelengths of 440 nm and 490 nm. Generalized polarization (GP) values were calculated using the formula GP = (I₄₄₀ − I₄₉₀)/(I₄₄₀ + I₄₉₀), where I₄₄₀ and I₄₉₀ represent the fluorescence intensities at 440 nm and 490 nm, respectively. Higher GP values indicate increased membrane order and decreased fluidity.

For membrane permeability analysis, PI fluorescence was measured at excitation/emission wavelengths of 535/615 nm, and NPN fluorescence was measured at 350/420 nm. Relative to the negative control, a significant increase in PI fluorescence indicated compromised inner membrane integrity, while enhanced NPN fluorescence reflected increased outer membrane permeability.

### Gene expression analysis via quantitative real-time PCR (qPCR)

HZ055, 055R, HZ055Δ*WaaL*, YW047, YW047Δ*WaaP*, and 055R2P strains were cultured to the logarithmic growth phase. Total bacterial RNA was extracted using a Vazyme RNA extraction kit following the manufacturer’s protocols. Concentration and purity were then quantified. Equal amounts of RNA were reverse-transcribed into first-strand cDNA using reverse transcriptase. Specific primers targeting the colistin resistance-related genes (*pmrA*, *pmrB*, *arnT*, *eptA*, *pmrD*, *pmrG*, and *pmrR*) and the reference gene *gyrB* were designed using Primer Premier 6 software (Additional file 3). qPCR reactions were prepared with cDNA as the template and run on a real-time fluorescence quantitative PCR instrument using a standard three-step protocol: pre-denaturation, followed by denaturation–annealing–extension cycles, and a final melting curve analysis to verify amplification specificity and efficiency. Relative gene expression levels were calculated using the 2^(–ΔΔ*C*_t_) method. Briefly, the cycle threshold (*C*_t_) values of target genes were firstly normalized to those of the reference gene *gyrB* to obtain Δ*C*_t_ values. Then, subsequent comparison of these Δ*C*_t_ values with those of the wild-type control group yielded the relative expression levels of each target gene.

### Statistical analysis

All analyses were performed with GraphPad Prism 6 and SPSS. Data are expressed as mean ± SD. Comparisons between two groups were evaluated with an unpaired *t*-test; comparisons among multiple groups were evaluated with one-way analysis of variance (ANOVA) followed by Tukey’s post hoc test. Statistical significance was set at *P < 0.05, **P < 0.01, and ***P < 0.001.

## Results

### Establishment of ITPFS and efficacy of dual-phage cocktail

**Figure 1:**
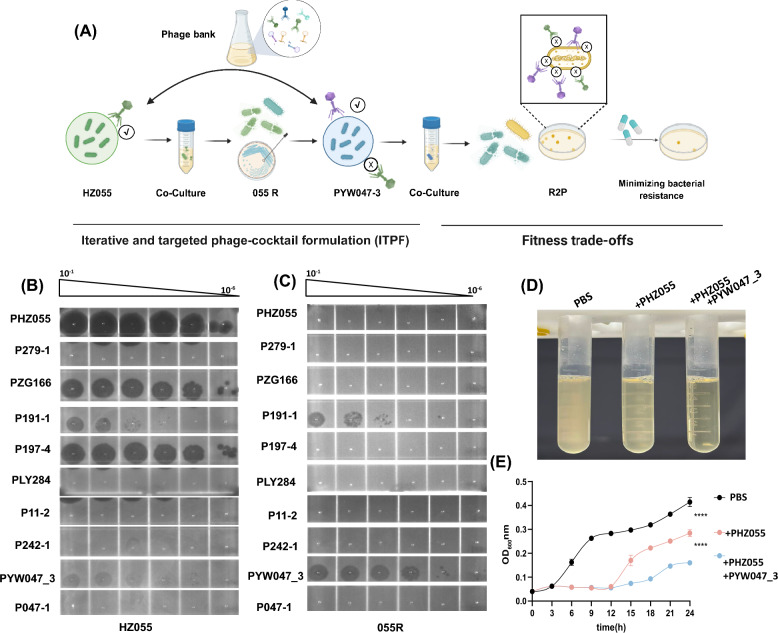
**Development of ITPFS and screening/validation of PHZ055 and PYW047_3.** **A** Workflow for the development of an ITPFS targeting the multidrug-resistant pathogenic *S.* Pullorum strain HZ055. **B** First-round screening: Lytic activity assay of the phage library against strain HZ055. **C** Second-round screening: Lytic activity assay of the phage library against the first-round resistant mutant 055R. **D**, **E** Comparison of the in vitro therapeutic efficacy of phage cocktails, evaluated via bacterial growth kinetics and test tube turbidity assays. Statistical significance was determined by one-way ANOVA [*P < 0.05, **P < 0.01, ***P < 0.001, ****P < 0.0001, *ns* not significant (*P* > 0.05)].

An iterative and targeted phage cocktail formulation strategy (ITPFS) was successfully developed and validated (Figure 1A) for the treatment of MDR pathogenic *S.* Pullorum strain HZ055, which was isolated from dead chicken embryos. Subsequently, 10 lytic phages with high lytic activity from our phage library were selected to perform interactive EOP assays against HZ055 (Additional file 4). Among these phages, one isolate (designated PHZ055) exhibited potent lytic activity against HZ055 even at a dilution of 10⁻⁶ PFU/mL (Figure 1B). This robust lytic capacity indicates excellent therapeutic potential, prompting its selection as the target phage for the first round of screening.

Given that rapid evolution of phage resistance in host bacteria severely compromises therapeutic efficacy, a second round of phage screening was conducted using the phage-resistant mutant 055R (evolved from HZ055). Results showed that phage PYW047_3 maintained strong lytic activity against 055R even at a dilution of 10⁻^5^ PFU/mL (Figure 1C), then PYW047_3 was selected as the target phage for the second round.

Notably, when the two phages were combined as a cocktail, results showed that bacterial growth was strongly suppressed and the emergence of resistance was markedly delayed relative to the single-phage PHZ055 treatment (Figure 1D, E).

### Morphology, biological, and genome analysis of PHZ055 and PYW047_3

**Figure 2:**
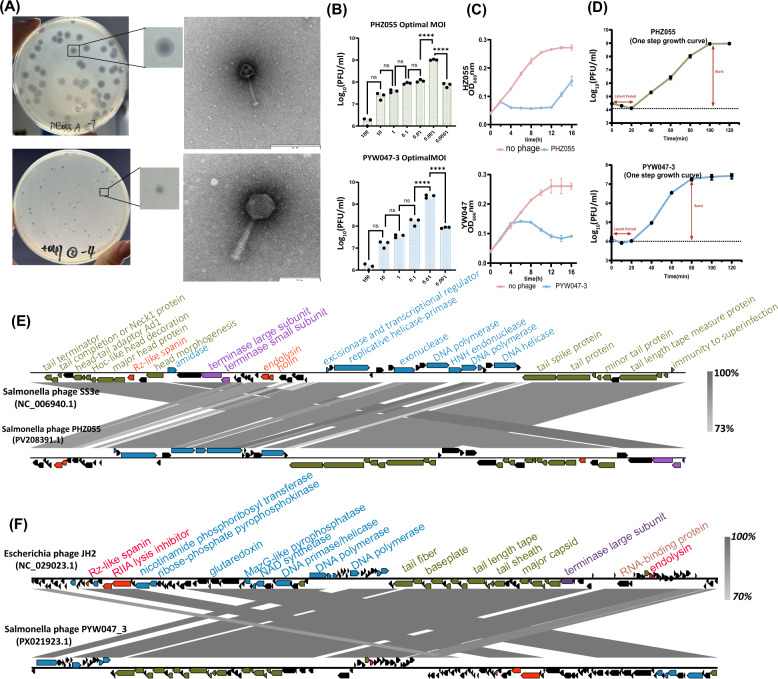
**Morphological, biological, and genomic analyses of PHZ055 and PYW047_3.** **A** Plaque morphology and transmission electron microscopy (TEM) images of PHZ055 (top panel) and PYW047_3 (bottom panel). **B** Determination of the optimal multiplicity of infection (MOI) for PHZ055 (top panel) and PYW047_3 (bottom panel). **C** Lytic kinetic assays of PHZ055 (top panel) and PYW047_3 (bottom panel). **D** One-step growth curves of PHZ055 (top panel) and PYW047_3 (bottom panel). **E** Genomic comparison between PHZ055 and phage SS3e. **F** Genomic comparison between PYW047_3 and phage JH2. Statistical significance was determined by one-way ANOVA [*P < 0.05, **P < 0.01, ***P < 0.001, ****P < 0.0001, *ns* not significant (*P* > 0.05)].

PHZ055 formed approximately 6-mm plaques with a central small regular circle and surrounding halo, implying the presence of genes encoding depolymerase. In contrast, PYW047_3 produced only about 1-mm plaques without a halo. Transmission electron microscopy (TEM) observations revealed distinct morphologies: PHZ055 was characterized as a siphovirus with a long, non-contractile tail (approximately 110 nm × 6 nm), while PYW047_3 was classified as a myovirus featuring a contractile tail with a visible sheath structure (Figure 2A). The most prominent difference was observed in the tail fibers; PHZ055 possessed a tail fiber cluster region, whereas PYW047_3 lacked this structure.

MOI assays demonstrated that the titers of phages PHZ055 and PYW047_3 reached their maximum when the phage-to-bacteria ratio was 0.001 and 0.01, respectively (Figure 2B). Growth kinetics analyses showed that host strain HZ055 evolved resistance to PHZ055 after 12 h. In contrast, no clear regrowth of YW047 was observed within the 16-h monitoring period following PYW047_3 treatment (Figure 2C). One-step growth curve assays indicated that both phages had a latent period of approximately 20 min. The burst size of PHZ055 was approximately 100 PFU/cell, calculated as the ratio of the plateau-phase titer (1 × 10⁹ PFU/mL at *t* = 100 min) to the initial bacterial concentration (1 × 10⁷ CFU/mL, MOI = 0.001). The burst size of PYW047_3 was approximately 20 PFU/cell (plateau-phase titer: 2 × 10⁷ PFU/mL at *t* = 80 min; initial bacterial concentration: 1 × 10⁶ CFU/mL, MOI = 0.01), indicating an approximately fivefold difference in burst size between the two phages (Figure 2D). Physicochemical stability assays demonstrated that both phages were sensitive to UV radiation (titer continuously decreased). They maintained viability at −80 to 37 °C and were stable at pH 5.0–9.0. Survival declined significantly at pH < 5.0 (Additional file 5).

Complete genomic sequencing showed that the two phages were genetically distinct and belonged to different phage genera. PHZ055 (40.82 kb with 60 predicted genes, accession no. PV208391.1) shared high similarity with phage SS3e (NC_006940.1) and could be classified into Genus Jerseyvirus (Figure 2E). PYW047_3 (87.615 kb with 132 predicted genes, accession no. PX021923.1) was highly homologous to phage JH2 (NC_029023.1) and then could be classified into Genus Felixounavirus. Notably, it encodes 18 tRNAs capable of actively transporting a diverse range of amino acids (Figure 2F). Neither phage contained AMR or toxin genes (Additional files 6 and 7). Whole-genome phylogenetic analysis of the two phages and related phages showed that *Escherichia* phages and *Salmonella* phages were interspersed in the same clusters, indicating that phages within the same genus may exhibit cross-sensitivity to *Enterobacteriaceae* (Additional files 8 and 9).

### Diverse mutations are identified in phage-resistant mutants

**Figure 3:**
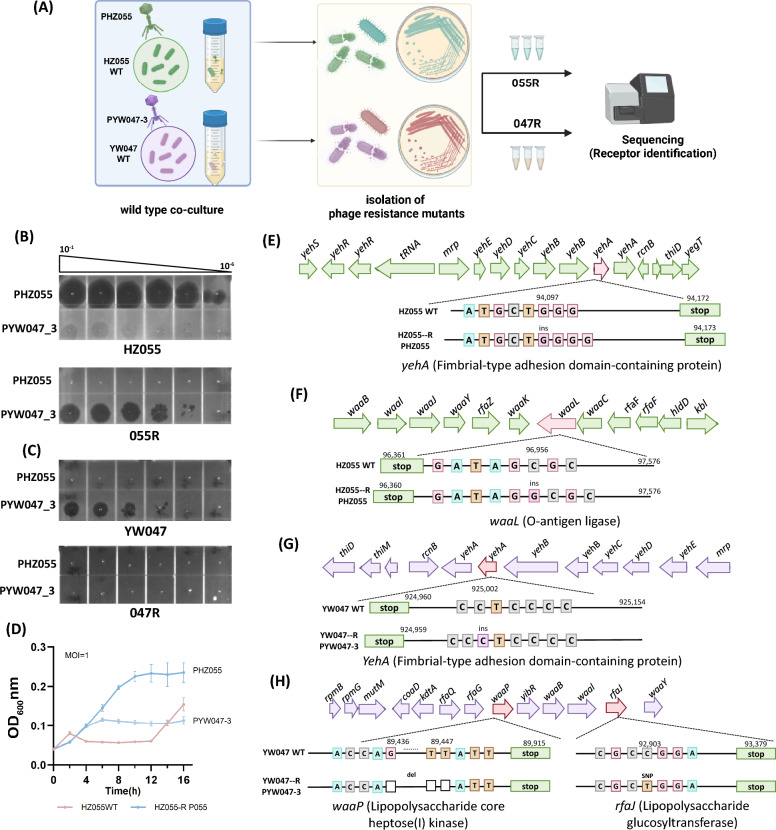
**Isolation, identification, and genomic comparative analysis of phage-resistant mutants.** **A** Schematic illustration of the workflow for isolation, identification, and genomic analysis of phage-resistant mutants. **B** Cross-plaque assays of the two phages against HZ055 (top panel) and its phage-resistant mutant 055R (bottom panel). **C** Cross-plaque assays of the two phages against YW047 (top panel) and its phage-resistant mutant 047R (bottom panel). **D** Lytic kinetic assays of PHZ055 and PYW047_3 against HZ055 (pink) and 055R (blue). **E** Single-nucleotide insertion mutation in the *YehA* gene of 055R. **F** Single-nucleotide insertion mutation in the *WaaL* gene of 055R. **G** Single-nucleotide insertion mutation in the *YehA* gene of 047R1. **H** Segment deletion mutation in the *WaaP* gene and missense mutation in the *rfaJ* gene of 047R.

Figure 3A depicts the schematic diagram for the identification and genome analysis of phage-resistant mutants. Cross-plaque assays between the two phages showed that PHZ055 lost the ability to lyse the PHZ055-resistant mutants 055R, whereas PYW047_3 formed distinct clear plaques (Figure 3B). Meanwhile, only PYW047_3 was capable of lysing its parental host strain YW047, and neither phage could lysis the PYW047_3-resistant mutants 047R (Figure 3C). Lytic kinetics experiments further confirmed that 055R had acquired complete resistance to PHZ055, while PYW047_3 effectively lysed 055R (Figure 3D). These results collectively demonstrate that the acquisition of resistance to PHZ055 conversely enhances susceptibility of 055R to PYW047_3.

To preliminarily explore the underlying resistance mechanism, genome analysis of the selected phage-resistant mutants was subsequently performed. Two PHZ055-resistant mutants (055R1–R2) and four PYW047_3 resistant mutants (047R1–R4) were aligned to the genomes of HZ055 and YW047, respectively. All the annotation results for the resistant mutants are summarized in Table 1. Both 055R mutants carried two identical mutation sites. One was localized in the gene *YehA* (a single-nucleotide insertion, TGGG → TGGGG), which encodes a bacterial surface protein typically containing a pilus-type adhesion domain (Figure 3E). The other was in the gene *WaaL* (a single-nucleotide insertion, GCGC → GGCGC). The *WaaL* encodes a key enzyme in bacterial lipopolysaccharide (LPS) biosynthesis, which is responsible for linking the O-antigen polysaccharide chain to the LPS core oligosaccharide (Figure 3F).

**Table 1:** **The name, mutation site and type of the mutant gene in the sequenced strain and the function encoded by the gene**

Mutants	Genes	Nucleotide change	Mutated amino acid position	Function
055R1	*YehA*	ins:(T → TG)	40	Fimbrial-type adhesion domain-containing protein
	*WaaL*	ins:(C → CG)	208	O-antigen ligase
	tRNA	coding(104267–104332/392223nt) SNP	–	tRNA
055R2	*YehA*	ins:(T → TG)	40	Fimbrial-type adhesion domain-containing protein
	*WaaL*	ins:(C → CG)	208	O-antigen ligase
047R1	*YehA*	ins:(T → TG)	40	Fimbrial-type adhesion domain-containing protein
	*WaaP*	del:(AGAACATCATTT → A)	107	Lipopolysaccharide core heptose(I) kinase WaaP
047R2	*YehA*	ins:(T → TG)	40	Fimbrial-type adhesion domain-containing protein
	*WaaP*	del:(AGAACATCATTT → A)	107	Lipopolysaccharide core heptose(I) kinase WaaP
	Hypothetical protein	SNP:(C → T)	96	Uncharacterized protein
047R3	*YehA*	ins:(T → TG)	40	Fimbrial-type adhesion domain-containing protein
	*WaaP*	del:(AGAACATCATTT → A)	107	Lipopolysaccharide core heptose(I) kinase WaaP
	*oadB1*	ins:(C → CG)	70	Oxaloacetate decarboxylase beta chain 1
047R4	*rfaJ*	SNP:(C → T)	108	Lipopolysaccharide glucosyltransferase RfaJ
	*oadA1*	SNP:(A → G)	–	Oxaloacetate decarboxylase alpha chain
	*bla*	SNP:(C → T)	–	Beta-lactamase
	JCM19231_3832	SNP:(A → G)	16	Beta-lactamase
	NPIL_447491	complex:(TTA…CCG → CTG…AAA)	–	Maturase K

Four 047R mutants shared two identical mutation sites: one also located in the gene *YehA* (single-nucleotide insertion, CCTCC → CCCTCC) (Figure 3G), and the other involved a segment deletion in the gene *WaaP* (AGAACATCATTTA → AA) and a missense mutation in the gene *rfaJ* (CCGG → CTGG) (Figure 3H). Gene *WaaP* encoding a key enzyme that phosphorylates the first heptose of the LPS core oligosaccharide.

### Receptor *WaaL* and *WaaP* mediate cross-lytic susceptibility

**Figure 4:**
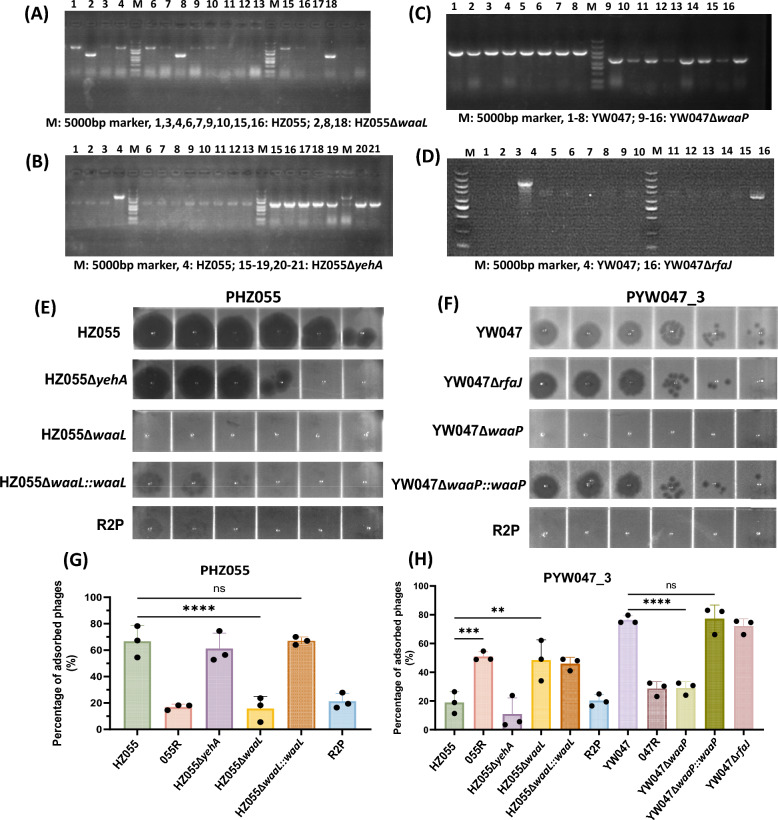
**Construction of gene knockout strains and their effects on phage plaque formation efficiency and adsorption efficiency**. **A**–**D** Construction and validation of *waaL*, *yehA*, *waaP*, and *rfaJ* knockout strains, respectively. **E** Plaque formation efficiency assay of PHZ055 against HZ055, HZ055Δ*yehA*, HZ055Δ*waaL*, HZ055Δ*waaL*::*waaL* (waaL-complemented strain) and R2P (top to bottom). **F** Plaque formation efficiency assay of PYW047_3 against YW047, YW047Δ*rfaJ*, YW047Δ*waaP*, YW047Δ*waaP*::*waaP* (waaP-complemented strain), R2P, and HZ055Δ*waaL* (top to bottom). **G** Adsorption efficiency of PHZ055 against HZ055, 055R, HZ055Δ*yehA*, HZ055Δ*waaL*, and R2P (left to right). **H** Adsorption efficiency of PYW047_3 against HZ055, 055R, HZ055Δ*yehA*, HZ055Δ*waaL*, R2P, YW047, 047R, YW047Δ*waaP*, and YW047Δ*rfaJ* (left to right). Statistical significance was determined by one-way ANOVA [*P < 0.05, **P < 0.01, ***P < 0.001, ****P < 0.0001, *ns* not significant (*P* > 0.05)].

To elucidate the host surface receptors of phages PHZ055 and PYW047_3, 0gene knockout mutants HZ055*ΔWaaL* (Figure 4A), HZ055*ΔyehA* (Figure 4B), YW047*ΔwaaP* (Figure 4C), and YW047*ΔrfaJ* (Figure 4D) were successfully constructed. EOP assays showed that deletion of the *yehA* gene in HZ055 reduced the infection efficiency of PHZ055 by only 100-fold, whereas deletion of *waaL* completely abolished susceptibility to the PHZ055 with infection efficiency dropping by approximately 10⁶-fold. Complementation of the *waaL* partially restored susceptibility to PHZ055 but was not fully restored in HZ055Δ*waaL*::*waaL* (Figure 4E). On the other hand, deletion of the *rfaJ* gene in YW047 reduced the infection efficiency of PYW047_3 by only tenfold, whereas deletion of *waaP* completely abolished susceptibility to PYW047_3 with infection efficiency dropping by approximately 10⁶-fold. Complementation of the *waaP* genes restored full susceptibility to PYW047_3 in YW047Δ*waaP*::*waaP* (Figure 4F).

Phage adsorption assays indicated that the adsorption efficiency of PHZ055 to 055R and HZ055Δ*waaL* was significantly decreased by approximately 50% compared to the host strain HZ055. Notably, adsorption of PHZ055 to the complemented strain HZ055Δ*waaL*::*waaL* was fully restored (Figure 4G). Similarly, the adsorption efficiency of PYW047_3 to 047R and YW047Δ*waaP* decreased by approximately 50% relative to host strain YW047. Complementation in YW047ΔwaaP::waaP fully restored adsorption. Furthermore, the adsorption efficiency of PYW047_3 to 055R and HZ055Δ*waaL* was significantly increased by about 40% compared to its adsorption to HZ055, which is consistent with the results of the EOP assays. Notably, the percentage of adsorbed PYW047_3 phages to the complemented strain HZ055Δ*waaL*::*waaL* remained significantly elevated compared to the parental strain HZ055, suggesting that *waaL* complementation did not fully restore O-antigen-mediated shielding of the inner core LPS region (Figure 4H). Collectively, these results demonstrated *WaaL* and *WaaP* served as the receptor for PHZ055 and PYW047_3, respectively. They also imply that the cross-lytic ability of PYW047_3 against the HZ055 strain likely originates from receptor mutations acquired by HZ055 to escape from PHZ055 infection.

### Combination of two phages incurs diverse adaptive trade-offs

**Figure 5:**
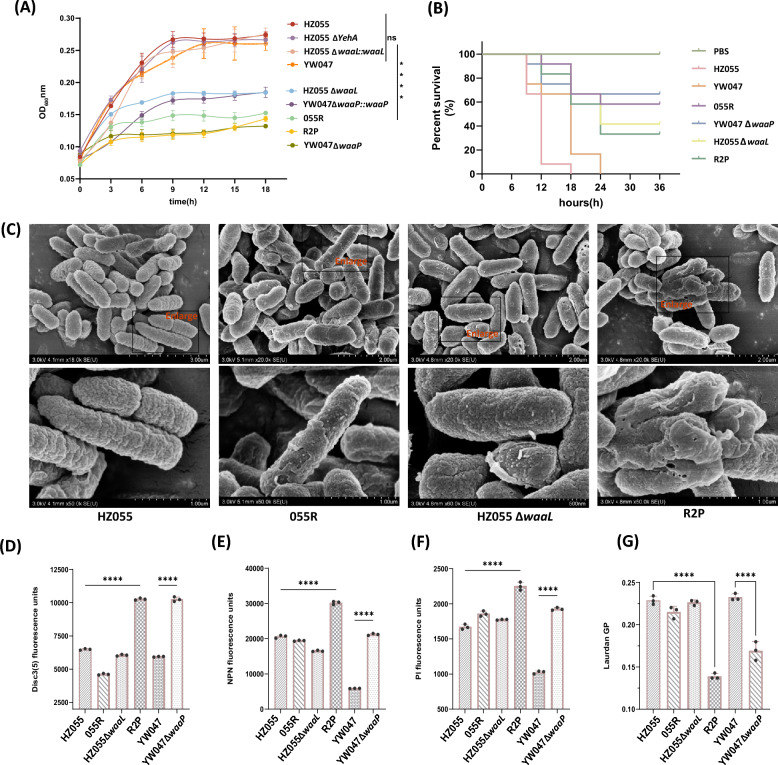
**Physiological fitness costs of phage-resistant bacteria**. **A** Growth curves of HZ055, HZ055Δ*yehA*, HZ055Δ*waaL*, 055R, and R2P. **B** Survival curves of *G. mellonella* infected with HZ055, YW047, HZ055Δ*waaL*, YW047Δ*waaP*, 055R, and R2P (*n* = 12). **C** Scanning electron microscopy (SEM) micrographs of HZ055, 055R, HZ055Δ*waaL,* and R2P (left to right). **D** plasma membrane potential determination of HZ055, 055R, HZ055Δ*waaL*, R2P, YW047, and YW047Δ*waaP*. **E** Plasma membrane permeability assays of HZ055, 055R, HZ055Δ*waaL*, R2P, YW047, and YW047Δ*waaP*. **F** Outer membrane permeability assays of HZ055, 055R, HZ055Δ*waaL*, R2P, YW047, and YW047Δ*waaP*. **G** Membrane fluidity analysis of HZ055, 055R, HZ055Δ*waaL*, R2P, YW047, and YW047Δ*waaP*. Statistical significance was determined by one-way ANOVA [*P < 0.05, **P < 0.01, ***P < 0.001, ****P < 0.0001, ns: not significant (*P* > 0.05)].

Growth kinetic analysis showed that compared to their respective parental strains, the growth levels of HZ055Δ*WaaL* and 055R were significantly lower than that of HZ055, and the growth level of YW047Δ*WaaP* was also significantly lower than that of YW047. The complemented strain HZ055Δ*WaaL*::*WaaL* fully recovered growth to the wild-type level. In contrast, YW047Δ*WaaP*::*WaaP* exhibited only partial growth restoration, suggesting that complementation of *WaaP* failed to fully rescue the corresponding fitness cost. Notably, among all tested strains, the R2P strain and the *WaaP*-deficient strain exhibited the most severe growth restriction, indicating profound growth defects (Figure 5A). In the *G. mellonella* infection model, survival rates dropped to 0% within 24 h following challenge with HZ055 or YW047. In contrast, challenged with HZ055Δ*WaaL*, YW047Δ*WaaP*, 055R, and R2P maintained survival rates above 30% even at 36 h post-challenge. Collectively, these findings indicate that the pathogenicity of phage-resistant mutants is significantly attenuated in vivo (Figure 5B).

SEM observation revealed distinct differences in surface morphology between strains. Under high resolution, HZ055 displayed a continuous, uniform, and intact envelope layer with a fine texture. In contrast, the surfaces of HZ055Δ*WaaL* and 055R were markedly compromised, characterized by increased roughness, extensive structural disruption, and pores of varying sizes. R2P exhibited not only these surface defects but also enhanced cell–cell adhesion along with partial depression or collapse of the cell surface (Figure 5C).

Comprehensive assessments of plasma membrane potential (Figure 5D), plasma membrane permeability (Figure 5E), and outer membrane permeability (Figure 5F) revealed distinct differences in membrane integrity across strains. Specifically, R2P and YW047Δ*waaP* exhibited marked membrane damage relative to their respective parental strains HZ055 and YW047. In contrast, 055R and HZ055Δ*WaaL* showed no significant deviations from the parental strain in these membrane-related parameters. Collectively, these findings imply that exposure to the PHZ055 and PYW047_3 phage cocktail elicits extensive membrane damage. Consistent with these observations, membrane fluidity analyses further confirmed that the phage combination-induced membrane dysfunction (Figure 5G).

### Collateral sensitivity to colistin and synergistic efficacy evaluation

**Figure 6:**
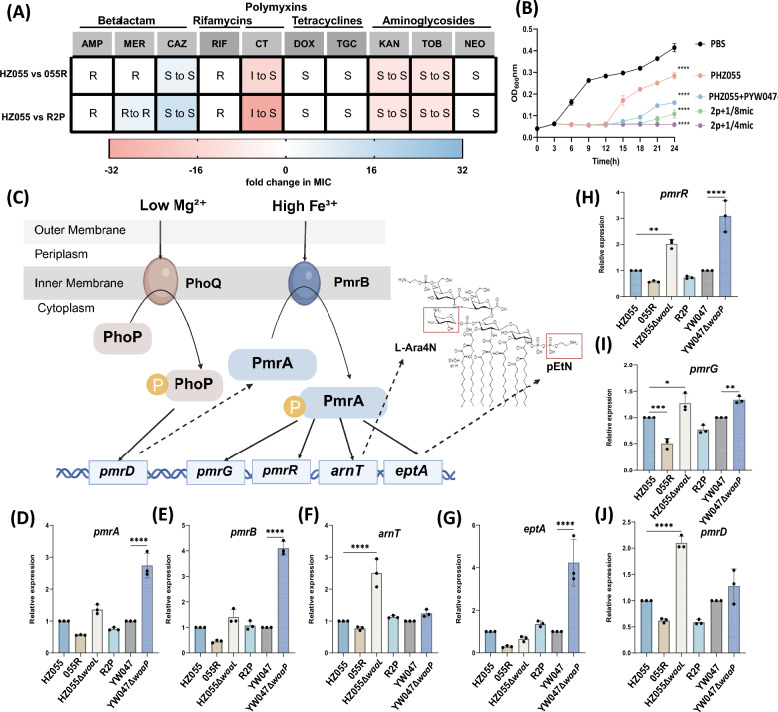
**Altered antibiotic susceptibility of phage-resistant strains and evaluation of synergistic phage-antibiotic therapy**. **A** Minimum inhibitory concentrations (MICs) of ten antibiotics against HZ055, 055R, and R2P determined via the broth microdilution method. Fold changes in MICs between HZ055 and 055R/R2P are indicated. *S* susceptible, *I* intermediate, *R* resistant. Antibiotics: *AMP* ampicillin, *MER* meropenem, *CAZ* ceftazidime, *RIF* rifampin, *CT* colistin, *DOX* doxycycline, *TGC* tigecycline, *KAN* kanamycin, *TOB* tobramycin, *NEO* neomycin. Color coding: red, decreased MIC; blue, increased MIC. Darker shades indicate a greater magnitude of change, while lighter shades indicate a smaller magnitude. **B** In vitro antibacterial efficacy of the phage cocktail combined with sub-inhibitory concentrations of colistin against HZ055. The combination of 2P (phage cocktail) + 1/4 MIC colistin (purple) exerted the strongest growth inhibitory effect. **C** Schematic illustration of the PmrA/B two-component system-mediated core regulatory pathway underlying colistin resistance. **D**–**J** Quantitative analysis of the expression levels of colistin resistance-related genes (*pmrA, pmrB, arnT, eptA, pmrD, pmrG, pmrR*) in different mutant strains. Statistical significance was determined by one-way ANOVA [*P < 0.05, **P < 0.01, ***P < 0.001, ****P < 0.0001, *ns* not significant (*P* > 0.05)].

Susceptibility of these mutant strains to a range of antimicrobials was detected (Table 2). Notably, 055R, 047R, R2P, and all strains exhibited increased collateral susceptibility to colistin. Compared with the parental strains HZ055 and YW047 (MIC = 2 µg/mL), the MIC values of 055R and HZ055Δ*waaL* were reduced fourfold (MIC = 0.5 µg/mL), whereas those of 047R, R2P, and YW047Δ*waaP* were decreased by 32-fold (MIC = 0.0625 µg/mL) (Figure 6A). To explore potential combinatorial therapeutic strategies, we evaluated the in vitro antibacterial efficacy of a phage cocktail (PHZ055 + PYW047_3, hereafter designated as 2P) in combination with sub-inhibitory concentrations of colistin (1/8 MIC and 1/4 MIC) against HZ055. The 2P + 1/8 MIC combination group exerted potent antibacterial activity. Remarkably, the 2P + 1/4 MIC colistin combination achieved the strongest growth inhibition effect (Figure 6B). Throughout the 24-h monitoring period, the OD₆₀₀ value of this combination group remained stable at an extremely low level, showing no significant difference from the initial inoculation baseline. This observation indicates complete suppression of bacterial regrowth. Survival curves analysis of the *G. mellonella* infection model further validated the synergistic therapeutic efficacy. The triple-combination group (PHZ055 + PYW047_3+ 1/4 MIC colistin) conferred the most robust synergistic protection, maintaining a survival rate above 70% at 24 h post-infection (Additional file 10). This survival benefit was significantly superior to that of all other treatment groups.

**Table 2:** **Minimum inhibitory concentration (MIC) of each antibiotic for each strain**

Strains	β-lactam				Aminoglycosides			Tetracyclines		Colistin	Quinolones		Sulfonamides	Glycopeptides	Rifamycins	Amphenicols
	Penicillins		Carbapenems	Cephalosporins												
	AMP	TLC	MEM	CAZ	TOB	KAN	NEO	DOX	TGC	CT	ENR	NOR	SMZ	VAN	RIF	FF
HZ055	256	128	8	0.5	2	2	2	0.125	< 0.25	2	0.5	0.5	256	256	4	4
055R	256	128	8	1	1	1	2	0.125	< 0.25	0.5	0.5	0.5	256	256	4	4
YW047	256	128	8	0.5	1	2	2	0.125	< 0.25	2	0.5	0.5	256	256	4	4
047R	256	128	16	1	1	2	1	0.125	< 0.25	0.0625	0.5	0.5	256	256	4	4
055Δ*YehA*	256	128	8	0.5	2	2	2	0.125	< 0.25	2	0.5	0.5	256	256	4	4
055Δ*WaaL*	256	128	8	0.5	1	4	2	0.125	< 0.25	0.5	0.5	0.5	256	256	4	4
047Δ*WaaP*	256	128	16	0.5	1	2	2	0.125	< 0.25	0.0625	0.5	0.5	256	256	4	4
R2P	256	128	8	2	1	1	2	0.125	< 0.25	0.0625	0.5	0.5	256	256	4	4

*AMP* ampicillin, *TLC* ticarcillin, *MEM* meropenem, *CAZ* ceftazidime, *TOB* tobramycin, *KAN* kanamycin, *NEO* neomycin, *DOX* doxycycline, *TGC* tigecycline, *CT* colistin, *ENR* enrofloxacin, *NOR* norfloxacin, *SMZ* sulfamethoxazole, *VAN* vancomycin, *RIF* rifampin, *FF* florfenicol.

The two-component system PmrAB serves as a central regulator of chromosome-mediated colistin resistance, while EptA and ArnT function as key enzymatic effectors that mediate lipid A modification (Figure 6C). To elucidate the molecular basis by which phage-resistance mutations enhance polymyxin susceptibility, we quantified the expression levels of colistin-related genes, including *pmrA, pmrB, arnT, eptA, pmrD, pmrG*, and *pmrR* (Figure 6D–J). Results revealed substantial variations in the expression of Pmr system genes across different mutant strains. Specifically, LPS structural defects (HZ055Δ*WaaL* and YW047Δ*WaaP*) strongly and specifically activate the expression of Pmr system-related genes. In HZ055Δ*WaaL*, *pmrD, pmrG, pmrR*, and *arnT* were markedly upregulated, whereas *pmrA* and *pmrB* exhibited only modest upregulation, while *eptA* expression was downregulated. In YW047Δ*WaaP*, *pmrA*, *pmrB*, *pmrR*, and *eptA* were upregulated by more than twofold, and all other tested genes also showed an upward trend. In striking contrast, the expression of key Pmr system genes remained generally stable in the two phage-resistant mutants (055R and R2P).

## Discussion

**Figure 7:**
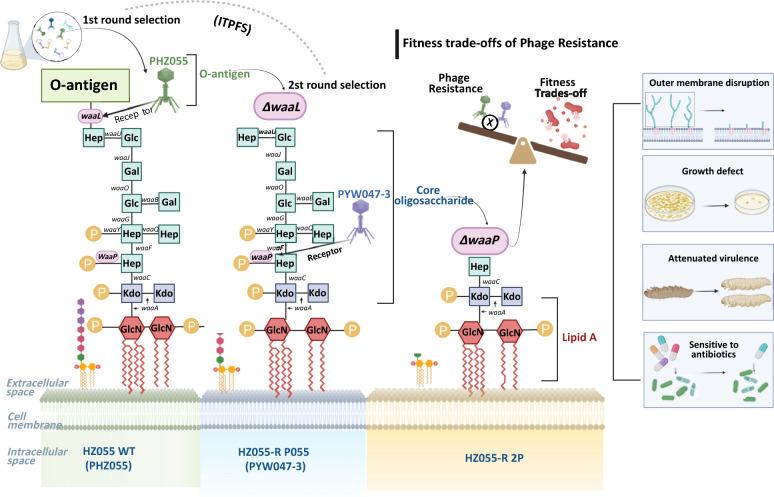
**Synergistic anti-MDR therapy via the ITPFS phage screening strategy**. This schematic systematically illustrates how the iterative temperate phage functional screening strategy (ITPFS) achieves highly effective synergistic therapy against the multidrug-resistant *S.* Pullorum strain HZ055. The efficacy is accomplished through iterative phage screening and the exploitation of fitness costs incurred by phage-resistant mutants. This strategy establishes a systematic framework for combating resistance in clinical phage therapy.

It is well known that the emergence of evolutionary phage resistance in target bacteria represents a major bottleneck limiting the clinical success of phage therapy [9]. To address this issue, a range of strategies has been proposed and experimentally demonstrated to enhance therapeutic outcomes, including phage training [45], phage cocktails [46], and phage–antibiotics synergy [47]. Meanwhile, a growing body of literature has discovered that evolved phage-resistant mutants exhibit diverse fitness costs, and exploiting these adaptive trade-offs has also been recognized as a promising approach to optimize phage therapy efficacy [10]. Nevertheless, such efforts remain scattered and have not yet been integrated into a targeted and systematic control framework for mitigating phage resistance in clinical settings. Herein, we reported and validated an iterative and targeted phage cocktail formulation strategy (ITPFS) that incorporates adaptive trade-offs to boost the therapeutic efficacy against MDR pathogenic *S.* Pullorum strain HZ055 (Figure 7).

Through two rounds of iterative selection, the lytic and targeted phages PHZ055 and PYW047_3 were successfully screened from our phage bank. Critical safety assessment via genomic annotation confirmed that neither PHZ055 nor PYW047_3 harbors any AMR genes or virulence factors, a key prerequisite for their potential application in clinical phage therapy [48, 49]. Morphological characterization integrated with genomic analysis revealed that these two phages belong to distinct phage genera. Further phylogenetic analysis demonstrated that *Escherichia* phages and *Salmonella* phages were interspersed within the same clades, suggesting that phages within the same genus may exhibit cross-genera host specificity toward Enterobacteriaceae, thus implying phage polyvalence. This observation indicates that the host range of these two phage genera is likely not strictly confined to a single bacterial species, which holds important implications for the rational design of broad-spectrum phage cocktails targeting clinically relevant co-occurring enteric pathogens [50].

Interestingly, a notable genomic feature of PYW047_3 is the presence of 18 tRNA genes. Theoretically, phages do not require tRNAs acquisition to assist protein translation, as they can hijack the host’s metabolic machinery to complete their own replication and assembly upon bacterial invasion [51]. However, recent study has described that the acquisition of tRNA genes represents an important adaptive strategy for phages during long-term coevolution with their hosts and confers multiple core advantages for efficient infection, replication, and survival [52]. Consistent with this, growth kinetics and one-step growth analyses showed that phage PYW047_3 poses better therapeutic potential, probably implying the critical role of phage-encoded tRNAs in enhancing phage performance.

Phage-encoded tRNA usually had two core roles, and translational efficiency optimization is the first core aspect. Phage genomes generally exhibit codon usage patterns distinct from their hosts, and these phage-borne tRNAs can precisely supplement the scarce tRNA species in host cells. This not only overcomes constraints of the host translational machinery but also accelerates phage gene translation and enhances viral particle assembly efficiency [53, 54]. Taking low-GC T4-like cyanophages for example, they can encode specialized tRNAs to accommodate their preferred low-GC codons and enable efficient proliferation in high-GC hosts without modifying their global genome GC content [53]. Also, phage-encoded tRNAs have been shown to act as important "weapons" against host antiviral immunity. From the host perspective, some hosts could block phage translation by degrading tRNAs, such as cleavage of tRNAs by VapC nucleases [55] or degradation of tRNA (Tyr) by the retron system [56], to suppress phage proliferation. In response, phage-encoded tRNAs can form cleavage resistance to offset the host's tRNA depletion to ensure the completion of the infection cycle. Although this host immune escape mechanism is not associated with enhanced lytic activity, the accelerated translation and improved assembly efficiency likely explain the functional differences between PYW047_3 and PHZ055. While further experimental evidence is required to validate these hypotheses, phage PYW047_3 nonetheless serves as a valuable model for future investigations into the functional mechanisms of phage-encoded tRNAs.

Receptor identification assays revealed that phages PHZ055 and PYW047_3 exploit lipopolysaccharide (LPS) as their receptor, whereas they recognized distinct LPS sites. Specifically, PHZ055 targets the O-antigen of LPS whose assembly is dependent on the *waaL* gene [57], while PYW047_3 recognizes the first heptose residue of the LPS inner core region whose formation is regulated by the *waaP* gene [58]. These findings are consistent with recent studies demonstrating that the enhanced efficacy of phage cocktails arises from their ability to target distinct receptors [29–31] and also confirmed our results that a combination of the two phages exerts effective suppression of bacterial growth. On the other hand, EOP assays showed that phage PYW047_3 formed clear plaques on both 055R and HZ055*ΔWaaL* strains, whereas only faint plaques were observed on the HZ055. Adsorption experiments further corroborated these EOP results. Collectively, these data indicate that mutation or deletion of the *waaL* gene exposes the LPS-binding epitope recognized by PYW047_3, thereby confirming the targeted efficacy of our screening strategy.

Typically, the *waaL* gene encodes O-antigen ligase, a conserved enzyme that catalyzes the final and crucial step in LPS maturation. It mediates the ligation of species-specific O-antigen polysaccharides to the lipid A–core oligosaccharide complex and is then indispensable for the surface display of O-antigen (receptor of PHZ055) on bacterial cells [57, 59-61]. At the same time, the *waaP* gene encodes a kinase that specifically phosphorylates the first heptose residues (receptor of PYW047_3) in the LPS core region, and thus its modification is critical for maintaining the structural integrity of the LPS core [58, 62]. Given the importance of these two genes for maintaining LPS integrity, we further explored the adaptive trade-offs when they mutated under pressure of our phage cocktail, and whether these costs could be exploited to further improve the therapeutic effectiveness of phages.

We found that strains 055R, HZ055Δ*WaaL*, and R2P all exhibited significant growth defects, reduced toxicity, and impaired cell wall structures, especially the resistant mutant R2P under PHZ055 and PYW047_3 pressure. Assays assessing extracellular and intracellular membrane permeability also further confirmed the existence of extensive adaptive costs. The most striking adaptive trade-off was that R2P exhibited a 32-fold increase in colistin sensitivity with MIC dropping from 2 to 0.0625. When the two phages were combined with 1/4 MIC colistin, bacterial growth was suppressed to near the detection limit within 24 h, demonstrating potent antibacterial synergy. Previous studies on *Salmonella typhimurium* have shown that *waaP*-mediated heptose phosphorylation is also involved in bacterial virulence and colistin resistance [58], indicating that *WaaP* represents a potential therapeutic target for phage therapy. To further elucidate the underlying mechanism of phage-induced collateral sensitivity to colistin, a series of chromosomally encoded colistin resistance-related regulatory genes were then qualified.

Usually, the development of colistin resistance driven by chromosomal modifications of lipid A is mediated by reducing its anionic charge and thus weakening colistin-LPS interactions [63]. Among the well-characterized chromosomal resistance pathways, the PmrAB two-component system serves as a central regulator, while EptA and ArnT are key enzymatic effectors of lipid A modification [64]. Specifically, ArnT- and EptA-controlled addition of positively charged residues 4-amino-(L)-arabinose (L-Ara4N) and phosphoethanolamine (pEtN) to LPS respectively results in a reduced negative charge on the bacterial surface and consequently increases the colistin resistance [65]. In the present study, MIC assays revealed consistent restoration of colistin susceptibility in both knockout strains (HZ055Δ*waaL* and YW047Δ*WaaP*) and phage evolved resistant mutants (055R and R2P). However, these two groups of strains differed fundamentally in the expression profiles of colistin resistance-related genes. The knockout strains exhibited significant upregulation of these genes, whereas the phage-resistant mutants showed slightly fluctuation in their expression. Notably, increased inner and outer membrane permeability was observed in these strains. This raises the question of whether the restoration of colistin susceptibility is mediated by enhanced membrane permeability, which would facilitate colistin access to its target sites [66]. Nevertheless, divergent mechanisms underlying susceptibility restoration must still be explained, and this process can also provide new insights into the modulatory factors of colistin susceptibility and offers potential targets for developing colistin resistance reversal strategies. We acknowledge that only a small number of resistant colonies were selected and sequenced to explore mutations linked to phage resistance. While the mutations we identified can well explain the mechanistic basis of resistance development, our limited sample size does not allow us to confirm whether these genetic changes are widespread. Further investigations with more resistant isolates will be necessary to fully clarify the overall mutation spectrum.

In conclusion, our reported and validated ITPFS indeed helped us screen the two phages PHZ055 and PYW047_3. The observation that *waaL* mutation eliminates PHZ055's receptor (O-antigen) while concomitantly exposing PYW047_3's receptor (heptose core) not only validates the specificity of our receptor identification but also clarifies the mechanistic basis of their interaction. This interactive pattern further supports the directional screening of phage cocktails composed of phages targeting these interactively regulated LPS epitopes. Additionally, the inevitable adaptive trade-offs (i.e., collateral sensitivity to colistin in this study) were also integrated and exploited to enhance the therapeutic efficacy. Critically, this strategy shows promising broad-spectrum applicability and may be readily extended to other clinically representative MDR bacterial isolates.

## Supplementary Information

**Additional file 1. Bacterial strains, phages and plasmids used in this study.**

**Additional file 2. All URL links originally appearing in the main text.**

**Additional file 3. Nucleotide sequences of primers used in this study.**

**Additional file 4. Lytic spectrum of PHZ055 and PYW047-3.**

**Additional file 5. Temperature, pH, UV, and chloroform stability of PHZ055 and PYW047-3. **(A, B) Phages were incubated at −80, −20, 4, 37, 50, and 60°C for 1 h to assess the thermostability of PHZ055 and PYW047-3. (C, D) Phages were incubated at 37°C for 1 h across a pH range of 1 to 13 to evaluate the pH tolerance of PHZ055 and PYW047-3. (E) Phages were separately exposed to ultraviolet (UV) irradiation, and samples were taken at regular intervals to determine the titer, in order to assess the UV sensitivity of PHZ055 and PYW047-3. (F) To evaluate the chloroform sensitivity of the phages, PHZ055 and PYW047-3 were separately co-incubated with chloroform, and the changes in titer before and after treatment were measured.

**Additional file 6. Circular representation of Salmonella Pullorum bacteriophage PHZ055.** The complete genome is 40.8 kb. Outer ring: predicted open reading frames (ORFs) colored by function. Inner ring: GC content (black) and GC skew (green/purple).

**Additional file 7. Circular representation of Salmonella Pullorum bacteriophage PYW047-3.** The complete genome is 87.6 kb. Outer ring: predicted open reading frames (ORFs) colored by function. Inner ring: GC content (black) and GC skew (green/purple).

**Additional file 8. Phylogenetic analysis of bacteriophage PHZ055 and comparative genomics with related phages.** Whole-genome comparison revealed that it shares the highest genomic homology with *Salmonella* phage SS3e (NC_006940.1), providing a basis for predicting its host range and supporting its taxonomic classification. Phylogenetic analysis indicates that bacteriophage PHZ055 belongs evolutionarily to the genus *Tunavirus*.

**Additional file 9. Phylogenetic analysis of bacteriophage PYW047-3 and comparative genomics with related phages.** Whole-genome comparison revealed that it shares the highest genomic homology with *Escherichia* phage JH-2 (NC_029023.1), providing a molecular basis for predicting its host range and supporting its taxonomic classification. Phylogenetic analysis indicates that bacteriophage PYW047-3 belongs evolutionarily to the genus *Felixounavirus*.

**Additional file 10. In vivo synergistic effect of combined therapy in a G. mellonella model.** The in vivo efficacy of phage–antibiotic combination therapy against strain HZ055 was evaluated using a *Galleria mellonella* infection model with six treatment groups.

## Data Availability

No datasets were generated or analyzed during the current study.

## References

[1] Hu Y, Wang Z, Qiang B, Xu Y, Chen X, Li Q, Jiao X (2019) Loss and gain in the evolution of the salmonella enterica serovar gallinarum biovar pullorum genome. mSphere 4:10 10.1128/mSphere.00627-18 PMC6449608 30944215

[2] Kipper D, De Carli S, de Souza Zanetti N, Mascitti AK, Kazantzi Fonseca AS, Ikuta N, Lunge VR (2024) Evolution and genomic profile of *Salmonella enterica* serovar Gallinarum biovar Pullorum isolates from Brazil. Avian Dis 68:2–9 38687101 10.1637/aviandiseases-D-23-00017

[3] Pan Z, Wang X, Zhang X, Geng S, Chen X, Pan W, Cong Q, Liu X, Jiao X, Liu X (2009) Changes in antimicrobial resistance among *Salmonella enterica* subspecies enterica serovar Pullorum isolates in China from 1962 to 2007. Vet Microbiol 136:387–392 19128897 10.1016/j.vetmic.2008.11.015

[4] Gast RK, Porter RE (2020) Salmonella Infections. John Wiley & Sons, Inc., Hoboken, pp 717–753

[5] Zhou X, Kang X, Zhou K, Yue M (2022) A global dataset for prevalence of Salmonella Gallinarum between 1945 and 2021. Sci Data 9:495 35963862 10.1038/s41597-022-01605-x PMC9376096

[6] Miller WR, Arias CA (2024) ESKAPE pathogens: antimicrobial resistance, epidemiology, clinical impact and therapeutics. Nat Rev Microbiol 22:598–616 38831030 10.1038/s41579-024-01054-w PMC13147291

[7] Skurnik M, Alkalay-Oren S, Boon M, Clokie M, Sicheritz-Pontén T, Dąbrowska K, Hatfull GF, Hazan R, Jalasvuori M, Kiljunen S, Lavigne R, Malik DJ, Nir-Paz R, Pirnay J-P (2025) Phage therapy. Nat Rev Methods Primers 5:9

[8] Li L, Zhang M, Cai Y, Liu Z, Tang Z, Zhang Z, Liu Y, Zhao C, Yu X, Petersen B, Sicheritz-Ponten T, Clokie MRJ, Liu Y (2025) Optimizing phage therapy for *Salmonella* Pullorum: dosage, timing, and pharmacokinetics define treatment efficacy in SPF chickens. Poult Sci 104:105643 40848485 10.1016/j.psj.2025.105643 PMC12398828

[9] Oromi-Bosch A, Antani JD, Turner PE (2023) Developing phage therapy that overcomes the evolution of bacterial resistance. Annu Rev Virol 10:503–524 37268007 10.1146/annurev-virology-012423-110530

[10] Mangalea MR, Duerkop BA (2020) Fitness trade-offs resulting from bacteriophage resistance potentiate synergistic antibacterial strategies. Infect Immun 88:10 10.1128/IAI.00926-19 PMC7309606 32094257

[11] Zeng Y, Li P, Liu S, Shen M, Liu Y, Zhou X (2024) *Salmonella enteritidis* acquires phage resistance through a point mutation in rfbD but loses some of its environmental adaptability. Vet Res 55:85 38970094 10.1186/s13567-024-01341-7 PMC11227202

[12] Li G, Shen M, Yang Y, Le S, Li M, Wang J, Zhao Y, Tan Y, Hu F, Lu S (2018) Adaptation of *Pseudomonas aeruginosa* to Phage PaP1 predation via O-antigen polymerase mutation. Front Microbiol 9:1170 29910791 10.3389/fmicb.2018.01170 PMC5992289

[13] Han P, Lin W, Fan H, Tong Y (2024) Characterization of phage evolution and phage resistance in drug-resistant *Stenotrophomonas **maltophilia*. J Virol 98:e0124923 38189285 10.1128/jvi.01249-23 PMC10878236

[14] Fu Y, Yin M, Cao L, Lu Y, Li Y, Zhang L (2025) Capsule mutations serve as a key strategy of phage resistance evolution of K54 hypervirulent Klebsiella pneumoniae. Commun Biol 8:257 39966630 10.1038/s42003-025-07687-8 PMC11836320

[15] Taylor VL, Fitzpatrick AD, Islam Z, Maxwell KL (2019) The diverse impacts of phage morons on bacterial fitness and virulence. Adv Virus Res 103:1–31 30635074 10.1016/bs.aivir.2018.08.001

[16] Gaborieau B, Delattre R, Adiba S, Clermont O, Denamur E, Ricard JD, Debarbieux L (2024) Variable fitness effects of bacteriophage resistance mutations in *Escherichia coli*: implications for phage therapy. J Virol 98:e0111324 39213164 10.1128/jvi.01113-24 PMC11495123

[17] Zulk JJ, Clark JR, Ottinger S, Ballard MB, Mejia ME, Mercado-Evans V, Heckmann ER, Sanchez BC, Trautner BW, Maresso AW, Patras KA (2022) Phage resistance accompanies reduced fitness of uropathogenic *Escherichia coli* in the urinary environment. mSphere 7:e0034522 35920561 10.1128/msphere.00345-22 PMC9429881

[18] DeBardeleben HK, Lysenko ES, Dalia AB, Weiser JN (2014) Tolerance of a phage element by *Streptococcus pneumoniae* leads to a fitness defect during colonization. J Bacteriol 196:2670–2680 24816604 10.1128/JB.01556-14 PMC4097588

[19] Majkowska-Skrobek G, Markwitz P, Sosnowska E, Lood C, Lavigne R, Drulis-Kawa Z (2021) The evolutionary trade-offs in phage-resistant *Klebsiella pneumoniae* entail cross-phage sensitization and loss of multidrug resistance. Environ Microbiol 23:7723–7740 33754440 10.1111/1462-2920.15476

[20] Gao D, Ji H, Wang L, Li X, Hu D, Zhao J, Wang S, Tao P, Li X, Qian P (2022) Fitness trade-offs in phage cocktail-resistant *Salmonella enterica* serovar Enteritidis results in increased antibiotic susceptibility and reduced virulence. Microbiol Spectr 10:e0291422 36165776 10.1128/spectrum.02914-22 PMC9603643

[21] Gordillo Altamirano F, Forsyth JH, Patwa R, Kostoulias X, Trim M, Subedi D, Archer SK, Morris FC, Oliveira C, Kielty L, Korneev D, O’Bryan MK, Lithgow TJ, Peleg AY, Barr JJ (2021) Bacteriophage-resistant Acinetobacter baumannii are resensitized to antimicrobials. Nat Microbiol 6:157–161 33432151 10.1038/s41564-020-00830-7

[22] Chen Q, Zhang F, Bai J, Che Q, Xiang L, Zhang Z, Wang Y, Sjoling A, Martin-Rodriguez AJ, Zhu B, Fu L, Zhou Y (2024) Bacteriophage-resistant carbapenem-resistant *Klebsiella pneumoniae* shows reduced antibiotic resistance and virulence. Int J Antimicrob Agents 64:107221 38810938 10.1016/j.ijantimicag.2024.107221

[23] Yu YS, Wang MZ, Ju LY, Li MC, Zhao MS, Deng H, Rensing C, Yang QE, Zhou SG (2025) Phage-mediated virulence loss and antimicrobial susceptibility in carbapenem-resistant. mBio 16:e02957 39714187 10.1128/mbio.02957-24 PMC11796411

[24] Strathdee SA, Hatfull GF, Mutalik VK, Schooley RT (2023) Phage therapy: from biological mechanisms to future directions. Cell 186:17–31 36608652 10.1016/j.cell.2022.11.017 PMC9827498

[25] Egido JE, Costa AR, Aparicio-Maldonado C, Haas PJ, Brouns SJJ (2022) Mechanisms and clinical importance of bacteriophage resistance. FEMS Microbiol Rev 46:fuab048 34558600 10.1093/femsre/fuab048 PMC8829019

[26] Hesse S, Rajaure M, Wall E, Johnson J, Bliskovsky V, Gottesman S, Adhya S (2020) Phage resistance in multidrug-resistant Klebsiella pneumoniae ST258 evolves via diverse mutations that culminate in impaired adsorption. mBio 11:10 10.1128/mBio.02530-19 PMC6989104 31992617

[27] Gurney J, Brown SP, Kaltz O, Hochberg ME (2020) Steering phages to combat bacterial pathogens. Trends Microbiol 28:85–94 31744662 10.1016/j.tim.2019.10.007 PMC6980653

[28] Mu Y, Song Y, Tian X, Ding Z, Yao S, Li Y, Wang C, Wei D, Vollmer W, Zhang G, Feng J (2025) Leveraging collateral sensitivity to counteract the evolution of bacteriophage resistance in bacteria. mLife 4:143–154 40313983 10.1002/mlf2.70003 PMC12042119

[29] Wang M, Wei J, Jiang L, Jiang L, Zhang J, He X, Ren Y, Wang Z, Sun Y, Wang Z (2023) Coevolutionary phage training and Joint application delays the emergence of phage resistance in Pseudomonas aeruginosa. Virus Evol 9:vead067 38089014 10.1093/ve/vead067 PMC10712906

[30] Kim S, Son B, Kim Y, Kim H, Nam G, Shin H, Ryu S (2024) Targeted dual-receptor phage cocktail against *Cronobacter sakazakii*: insights into phage-host interactions and resistance mechanisms. Front Microbiol 15:1468686 39712890 10.3389/fmicb.2024.1468686 PMC11659082

[31] Borin JM, Avrani S, Barrick JE, Petrie KL, Meyer JR (2021) Coevolutionary phage training leads to greater bacterial suppression and delays the evolution of phage resistance. Proc Natl Acad Sci U S A 118:e2104592118 34083444 10.1073/pnas.2104592118 PMC8201913

[32] Liu Z, Tan X, Xiong M, Lu S, Yang Y, Zhu H, Zhang J, Luo X, Zhou C, Wei S, Zhou N, Liu X, Bai C, Pan Y, Ma Y (2025) Efficacy of precisely tailored phage cocktails targeting carbapenem-resistant *Acinetobacter baumannii* reveals evolutionary trade-offs: a proof-of-concept study. EBioMedicine 120:105942 40972224 10.1016/j.ebiom.2025.105942 PMC12478281

[33] Kunisch F, Campobasso C, Wagemans J, Yildirim S, Chan BK, Schaudinn C, Lavigne R, Turner PE, Raschke MJ, Trampuz A, Gonzalez Moreno M (2024) Targeting Pseudomonas aeruginosa biofilm with an evolutionary trained bacteriophage cocktail exploiting phage resistance trade-offs. Nat Commun 15:8572 39362854 10.1038/s41467-024-52595-w PMC11450229

[34] Darby EM, Trampari E, Siasat P, Gaya MS, Alav I, Webber MA, Blair JMA (2023) Molecular mechanisms of antibiotic resistance revisited. Nat Rev Microbiol 21:280–295 36411397 10.1038/s41579-022-00820-y

[35] Segall AM, Roach DR, Strathdee SA (2019) Stronger together? Perspectives on phage-antibiotic synergy in clinical applications of phage therapy. Curr Opin Microbiol 51:46–50 31226502 10.1016/j.mib.2019.03.005

[36] Wang M, Zhu H, Wei J, Jiang L, Jiang L, Liu Z, Li R, Wang Z (2023) Uncovering the determinants of model *Escherichia coli* strain C600 susceptibility and resistance to lytic T4-like and T7-like phage. Virus Res 325:199048 36681192 10.1016/j.virusres.2023.199048 PMC10194157

[37] Gurney J, Pradier L, Griffin JS, Gougat-Barbera C, Chan BK, Turner PE, Kaltz O, Hochberg ME (2020) Phage steering of antibiotic-resistance evolution in the bacterial pathogen, *Pseudomonas aeruginosa*. Evol Med Public Health 2020:148–157 34254028 10.1093/emph/eoaa026 PMC7547624

[38] Oyejobi GK, Xiong D, Shi M, Zhang X, Yang H, Xue H, Ogolla F, Wei H (2022) Genetic signatures from adaptation of bacteria to lytic phage identify potential agents to aid phage-killing of multidrug-resistant *Acinetobacter baumannii*. J Bacteriol 204:e00593-21 35156836 10.1128/jb.00593-21 PMC8923232

[39] Wang M, Zhang J, Wei J, Jiang L, Jiang L, Sun Y, Zeng Z, Wang Z (2024) Phage-inspired strategies to combat antibacterial resistance. Crit Rev Microbiol 50:196–211 38400715 10.1080/1040841X.2023.2181056

[40] Jiang L, Zhu H, Wei J, Jiang L, Li Y, Li R, Wang Z, Wang M (2023) *Enterobacteriaceae* genome-wide analysis reveals roles for P1-like phage-plasmids in transmission of *mcr-1*, *tetX4* and other antibiotic resistance genes. Genomics 115:110572 36746220 10.1016/j.ygeno.2023.110572

[41] Thurber RV, Haynes M, Breitbart M, Wegley L, Rohwer F (2009) Laboratory procedures to generate viral metagenomes. Nat Protoc 4:470–483 19300441 10.1038/nprot.2009.10

[42] Li R, Lu X, Peng K, Liu Z, Li Y, Liu Y, Xiao X, Wang Z (2020) Deciphering the structural diversity and classification of the mobile tigecycline resistance gene tet(X)-bearing plasmidome among bacteria. mSystems 5:10 10.1128/mSystems.00134-20 PMC7190383 32345737

[43] Bankevich A, Nurk S, Antipov D, Gurevich AA, Dvorkin M, Kulikov AS, Lesin VM, Nikolenko SI, Pham S, Prjibelski AD, Pyshkin AV, Sirotkin AV, Vyahhi N, Tesler G, Alekseyev MA, Pevzner PA (2012) SPAdes: a new genome assembly algorithm and its applications to single-cell sequencing. J Comput Biol 19:455–477 22506599 10.1089/cmb.2012.0021 PMC3342519

[44] Carvalhaes CG, Klauer AL, Rhomberg PR, Pfaller MA, Castanheira M (2022) Evaluation of Rezafungin provisional CLSI clinical breakpoints and epidemiological cutoff values tested against a worldwide collection of contemporaneous invasive fungal isolates (2019 to 2020. J Clin Microbiol 60:e0244921 35249367 10.1128/jcm.02449-21 PMC9020363

[45] Torriero-Smith N, Rogers BA, McDonald MJ, Barr JJ (2025) Harnessing “phage training” to bolster the therapeutic potential of bacteriophages. Curr Opin Microbiol 89:102696 41412056 10.1016/j.mib.2025.102696

[46] Subedi D, Gordillo Altamirano F, Deehan R, Perera A, Patwa R, Kostoulias X, Korneev D, Blakeway L, Macesic N, Peleg AY, Barr JJ (2025) Rational design of a hospital-specific phage cocktail to treat *Enterobacter cloacae* complex infections. Nat Microbiol 10:2702–2719 40993246 10.1038/s41564-025-02130-4 PMC12578640

[47] Kunz Coyne AJ, Eshaya M, Bleick C, Vader S, Biswas B, Wilson M, Deschenes MV, Alexander J, Lehman SM, Rybak MJ (2024) Exploring synergistic and antagonistic interactions in phage-antibiotic combinations against ESKAPE pathogens. Microbiol Spectr 12:e0042724 39082827 10.1128/spectrum.00427-24 PMC11468199

[48] Suh GA, Lodise TP, Tamma PD, Knisely JM, Alexander J, Aslam S, Barton KD, Bizzell E, Totten KMC, Campbell JL, Chan BK, Cunningham SA, Goodman KE, Greenwood-Quaintance KE, Harris AD, Hesse S, Maresso A, Nussenblatt V, Pride D, Rybak MJ, Sund Z, van Duin D, van Tyne D, Patel R, Antibacterial Resistance Leadership G (2022) Considerations for the use of phage therapy in clinical practice. Antimicrob Agents Chemother 66:e0207121 35041506 10.1128/aac.02071-21 PMC8923208

[49] Gordillo Altamirano FL, Barr JJ (2019) Phage therapy in the postantibiotic era. Clin Microbiol Rev 32:10 10.1128/CMR.00066-18 PMC6431132 30651225

[50] Yu P, Mathieu J, Yang Y, Alvarez PJJ (2017) Suppression of enteric bacteria by bacteriophages: importance of phage polyvalence in the presence of soil bacteria. Environ Sci Technol 51:5270–5278 28414441 10.1021/acs.est.7b00529

[51] Silva MD, Pinto G, Franca A, Azeredo J, Melo LDR (2024) Phage SEP1 hijacks Staphylococcus epidermidis stationary cells’ metabolism to replicate. mSystems 9:e0026324 38904376 10.1128/msystems.00263-24 PMC11265418

[52] van den Berg DF, Brouns SJJ (2025) Phage tRNAs: decoding the enigma. Trends Microbiol 33:1121–1131 40533310 10.1016/j.tim.2025.05.009

[53] Limor-Waisberg K, Carmi A, Scherz A, Pilpel Y, Furman I (2011) Specialization versus adaptation: two strategies employed by cyanophages to enhance their translation efficiencies. Nucleic Acids Res 39:6016–6028 21470965 10.1093/nar/gkr169 PMC3152331

[54] Bailly-Bechet M, Vergassola M, Rocha E (2007) Causes for the intriguing presence of tRNAs in phages. Genome Res 17:1486–1495 17785533 10.1101/gr.6649807 PMC1987346

[55] van den Berg DF, van der Steen BA, Costa AR, Brouns SJJ (2023) Phage tRNAs evade tRNA-targeting host defenses through anticodon loop mutations. eLife 12:e85183 37266569 10.7554/eLife.85183 PMC10264068

[56] Azam AH, Kondo K, Chihara K, Nakamura T, Ojima S, Nie W, Tamura A, Yamashita W, Sugawara Y, Sugai M, Cui L, Takahashi Y, Watashi K, Kiga K (2024) Evasion of antiviral bacterial immunity by phage tRNAs. Nat Commun 15:9586 39528469 10.1038/s41467-024-53789-y PMC11555353

[57] Han Y, Han X, Wang S, Meng Q, Zhang Y, Ding C, Yu S (2014) The *waaL* gene is involved in lipopolysaccharide synthesis and plays a role on the bacterial pathogenesis of avian pathogenic *Escherichia coli*. Vet Microbiol 172:486–491 24970366 10.1016/j.vetmic.2014.05.029

[58] Yethon JA, Gunn JS, Ernst RK, Miller SI, Laroche L, Malo D, Whitfield C (2000) *Salmonella enterica* serovar typhimurium *waaP* mutants show increased susceptibility to polymyxin and loss of virulence In vivo. Infect Immun 68:4485–4491 10899846 10.1128/iai.68.8.4485-4491.2000 PMC98355

[59] Yethon JA, Heinrichs DE, Monteiro MA, Perry MB, Whitfield C (1998) Involvement of *waaY*, *waaQ*, and *waaP* in the modification of *Escherichia coli* lipopolysaccharide and their role in the formation of a stable outer membrane. J Biol Chem 273:26310–26316 9756860 10.1074/jbc.273.41.26310

[60] Berry MC, McGhee GC, Zhao Y, Sundin GW (2009) Effect of a *waaL* mutation on lipopolysaccharide composition, oxidative stress survival, and virulence in *Erwinia amylovora*. FEMS Microbiol Lett 291:80–87 19076232 10.1111/j.1574-6968.2008.01438.x

[61] Morgenstein RM, Clemmer KM, Rather PN (2010) Loss of the *waaL* O-antigen ligase prevents surface activation of the flagellar gene cascade in *Proteus mirabilis*. J Bacteriol 192:3213–3221 20382766 10.1128/JB.00196-10 PMC2901702

[62] Zhao X, Lam JS (2002) WaaP of *Pseudomonas aeruginosa* is a novel eukaryotic type protein-tyrosine kinase as well as a sugar kinase essential for the biosynthesis of core lipopolysaccharide. J Biol Chem 277:4722–4730 11741974 10.1074/jbc.M107803200

[63] Moffatt JH, Harper M, Boyce JD (2019) Mechanisms of polymyxin resistance. Adv Exp Med Biol 1145:55–71 31364071 10.1007/978-3-030-16373-0_5

[64] Chen HD, Groisman EA (2013) The biology of the PmrA/PmrB two-component system: the major regulator of lipopolysaccharide modifications. Annu Rev Microbiol 67:83–112 23799815 10.1146/annurev-micro-092412-155751 PMC8381567

[65] Zhang H, Srinivas S, Xu Y, Wei W, Feng Y (2019) Genetic and biochemical mechanisms for bacterial lipid A modifiers associated with polymyxin resistance. Trends Biochem Sci 44:973–988 31279652 10.1016/j.tibs.2019.06.002

[66] Ashworth EA, Wright RCT, Shears RK, Wong JKL, Hassan A, Hall JPJ, Kadioglu A, Fothergill JL (2024) Exploiting lung adaptation and phage steering to clear pan-resistant *Pseudomonas aeruginosa* infections in vivo. Nat Commun 15:1547 38378698 10.1038/s41467-024-45785-z PMC10879199

