# Supplementary material for: WaaL–WaaP receptor cross-talk drives phage-induced collateral sensitivity to colistin in Salmonella Pullorum
Source: Vet Res. 2026 Aug 12;57:147. doi: 10.1186/s13567-026-01825-8 (PMC13471373; doi:10.1186/s13567-026-01825-8)
Supplement: Supplementary file 1 [file 13567_2026_1825_MOESM10_ESM.pdf]

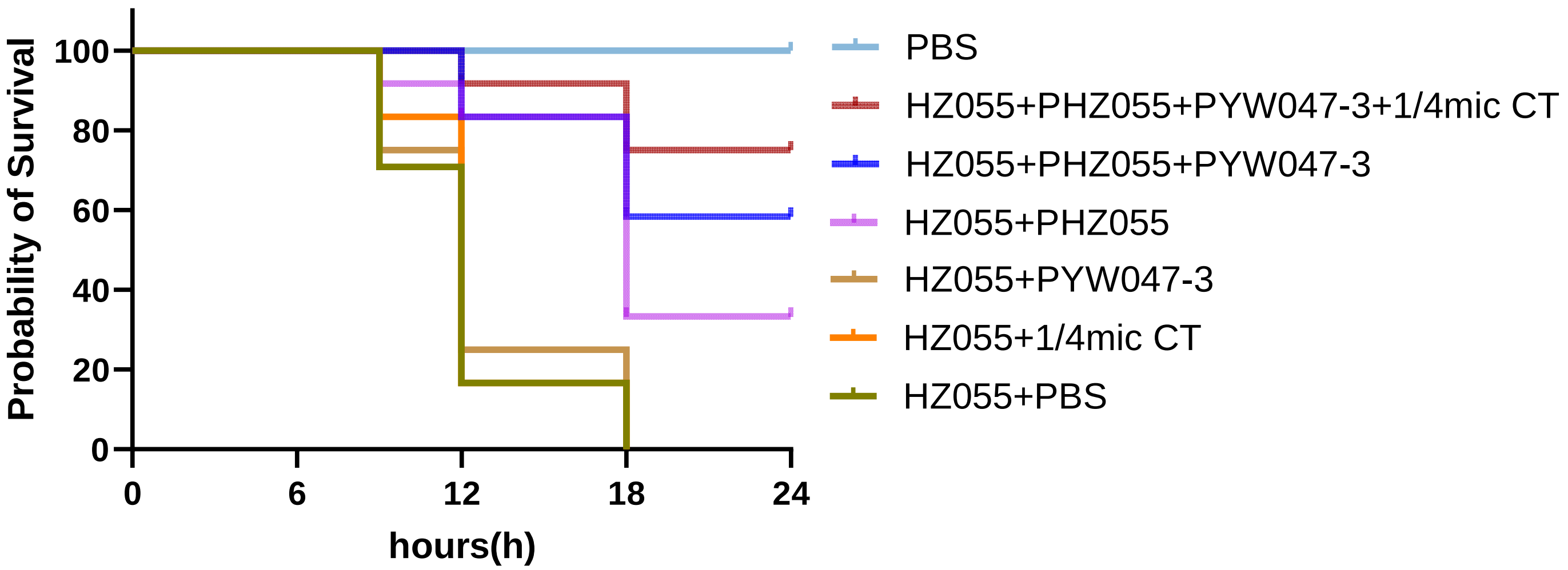

Probability of Survival
100
80
60
40
20
0
0
6
12
18
24
hours(h)
PBS
HZ055+PHZ055+PYW047-3+1/4mic CT
HZ055+PHZ055+PYW047-3
HZ055+PHZ055
HZ055+PYW047-3
HZ055+1/4mic CT
HZ055+PBS
