# Supplementary material for: WaaL–WaaP receptor cross-talk drives phage-induced collateral sensitivity to colistin in Salmonella Pullorum
Source: Vet Res. 2026 Aug 12;57:147. doi: 10.1186/s13567-026-01825-8 (PMC13471373; doi:10.1186/s13567-026-01825-8)
Supplement: Supplementary file 2 [file 13567_2026_1825_MOESM5_ESM.pdf]

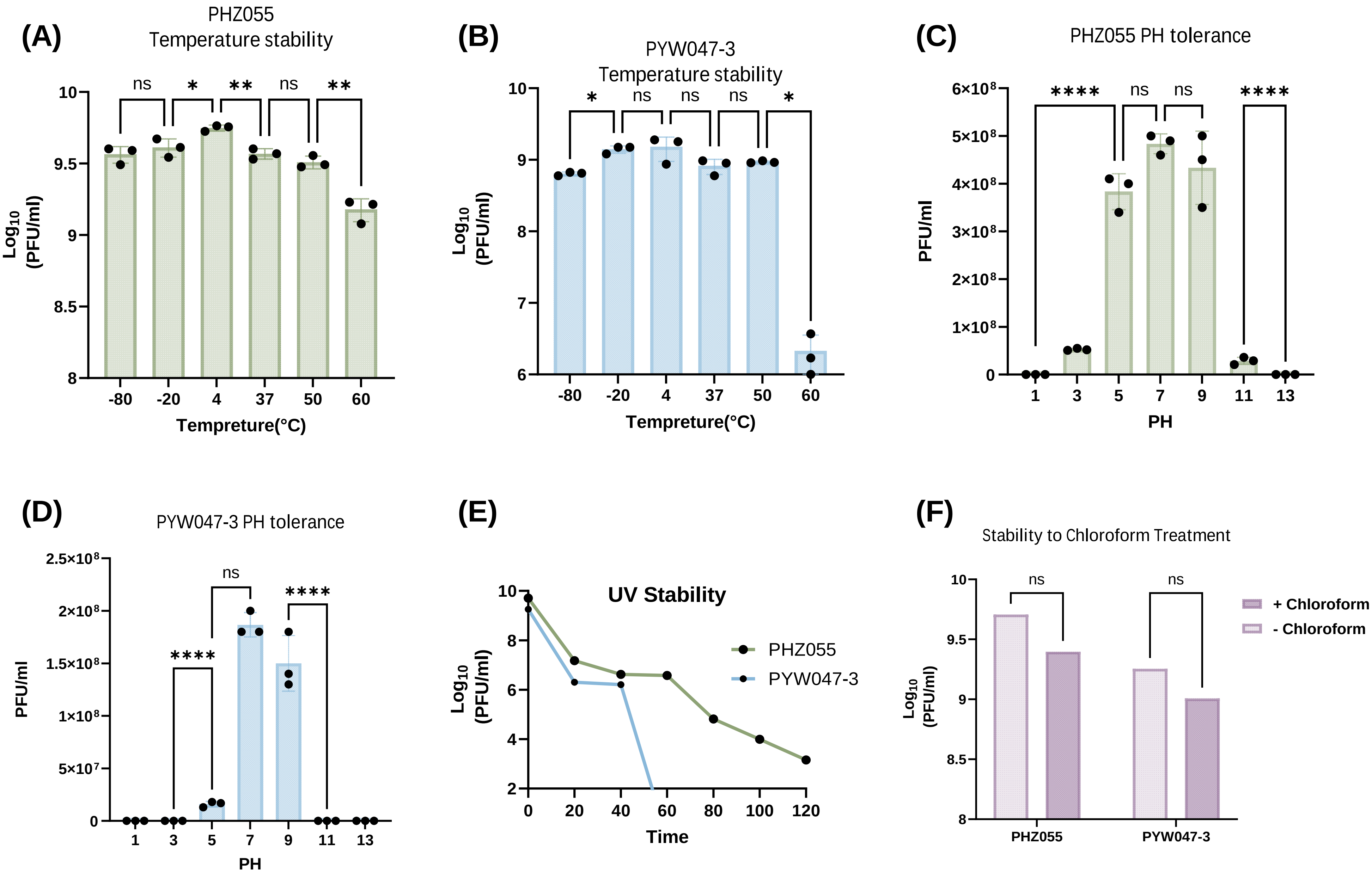

(A)
PHZ055
Temperature stability
ns
*
**
ns
**
Log10 (PFU/ml)
10
9.5
9
8.5
8
-80
-20
4
37
50
60
Tempreture(°C)
(B)
PYW047-3
Temperature stability
*
ns
ns
ns
*
Log10 (PFU/ml)
10
9
8
7
6
-80
-20
4
37
50
60
Tempreture(°C)
(C)
PHZ055 PH tolerance
****
ns
ns
****
PFU/ml
6×10⁸
5×10⁸
4×10⁸
3×10⁸
2×10⁸
1×10⁸
0
1
3
5
7
9
11
13
PH
(D)
PYW047-3 PH tolerance
ns
****
****
PFU/ml
2.5×10⁸
2×10⁸
1.5×10⁸
1×10⁸
5×10⁷
0
1
3
5
7
9
11
13
PH
(E)
UV Stability
PHZ055
PYW047-3
Log10 (PFU/ml)
10
8
6
4
2
0
20
40
60
80
100
120
Time
(F)
Stability to Chloroform Treatment
ns
ns
+ Chloroform
- Chloroform
Log10 (PFU/ml)
10
9.5
9
8.5
8
PHZ055
PYW047-3
