# Supplementary material for: WaaL–WaaP receptor cross-talk drives phage-induced collateral sensitivity to colistin in Salmonella Pullorum
Source: Vet Res. 2026 Aug 12;57:147. doi: 10.1186/s13567-026-01825-8 (PMC13471373; doi:10.1186/s13567-026-01825-8)
Supplement: Supplementary file 3 [file 13567_2026_1825_MOESM6_ESM.pdf]

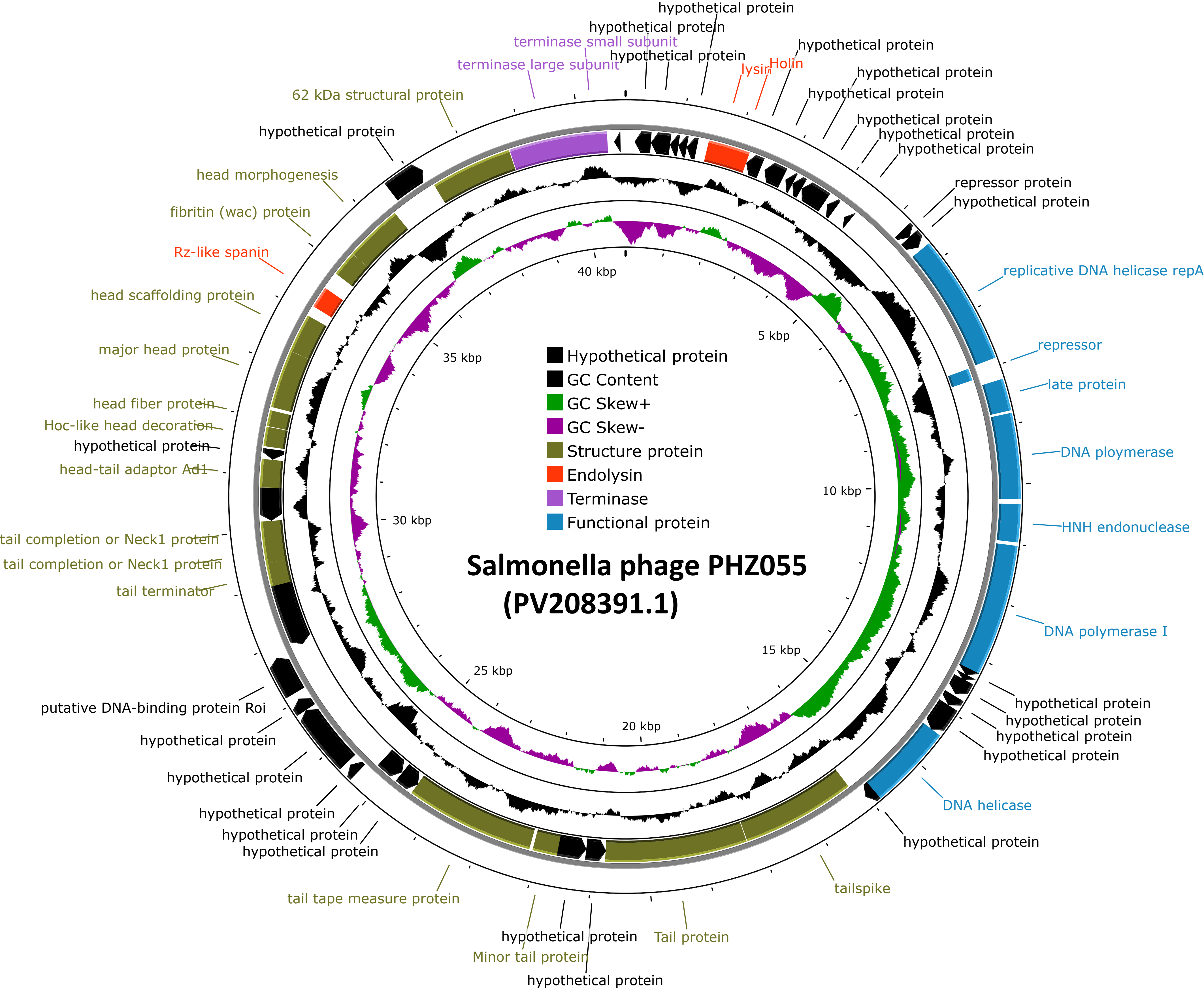

hypothetical protein
hypothetical protein
terminase small subunit
hypothetical protein
terminase large subunit
lysin
Holin
hypothetical protein
hypothetical protein
hypothetical protein
62 kDa structural protein
hypothetical protein
hypothetical protein
hypothetical protein
hypothetical protein
head morphogenesis
repressor protein
hypothetical protein
fibritin (wac) protein
Rz-like spanin
replicative DNA helicase repA
head scaffolding protein
major head protein
repressor
late protein
head fiber protein
Hoc-like head decoration
hypothetical protein
DNA ploymerase
head-tail adaptor Ad1
HNH endonuclease
tail completion or Neck1 protein
tail completion or Neck1 protein
tail terminator
DNA polymerase I
putative DNA-binding protein Roi
hypothetical protein
hypothetical protein
hypothetical protein
hypothetical protein
hypothetical protein
hypothetical protein
hypothetical protein
DNA helicase
hypothetical protein
hypothetical protein
hypothetical protein
tailspike
tail tape measure protein
hypothetical protein
Tail protein
Minor tail protein
hypothetical protein
40 kbp
5 kbp
35 kbp
10 kbp
30 kbp
15 kbp
25 kbp
20 kbp
Hypothetical protein
GC Content
GC Skew+
GC Skew-
Structure protein
Endolysin
Terminase
Functional protein
Salmonella phage PHZ055
(PV208391.1)
