# Supplementary material for: WaaL–WaaP receptor cross-talk drives phage-induced collateral sensitivity to colistin in Salmonella Pullorum
Source: Vet Res. 2026 Aug 12;57:147. doi: 10.1186/s13567-026-01825-8 (PMC13471373; doi:10.1186/s13567-026-01825-8)
Supplement: Supplementary file 4 [file 13567_2026_1825_MOESM7_ESM.pdf]

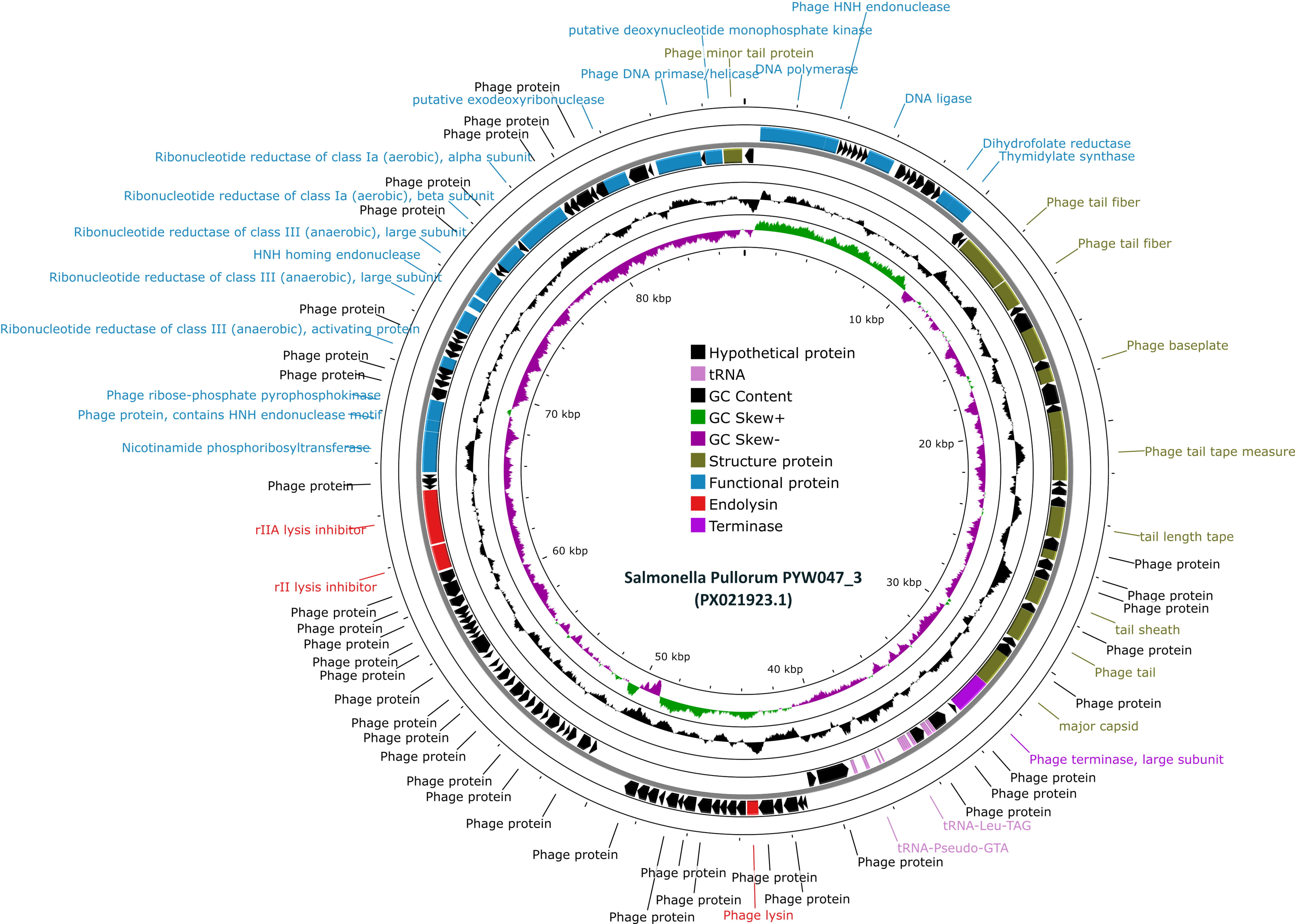
Phage HNH endonuclease
putative deoxynucleotide monophosphate kinase
Phage minor tail protein
Phage DNA primase/helicase
DNA polymerase
Phage protein
putative exodeoxyribonuclease
DNA ligase
Phage protein
Phage protein
Dihydrofolate reductase
Thymidylate synthase
Ribonucleotide reductase of class Ia (aerobic), alpha subunit
Phage protein
Ribonucleotide reductase of class Ia (aerobic), beta subunit
Phage tail fiber
Phage protein
Ribonucleotide reductase of class III (anaerobic), large subunit
Phage tail fiber
HNH homing endonuclease
Ribonucleotide reductase of class III (anaerobic), large subunit
Phage protein
Ribonucleotide reductase of class III (anaerobic), activating protein
Phage baseplate
Phage protein
Phage protein
Phage ribose-phosphate pyrophosphokinase
Phage protein, contains HNH endonuclease motif
Nicotinamide phosphoribosyltransferase
Phage tail tape measure
Phage protein
rIIA lysis inhibitor
tail length tape
Phage protein
rII lysis inhibitor
Phage protein
Phage protein
Phage protein
Phage protein
Phage protein
tail sheath
Phage protein
Phage protein
Phage protein
Phage tail
Phage protein
Phage protein
Phage protein
major capsid
Phage protein
Phage terminase, large subunit
Phage protein
Phage protein
Phage protein
Phage protein
Phage protein
tRNA-Leu-TAG
Phage protein
tRNA-Pseudo-GTA
Phage protein
Phage protein
Phage protein
Phage protein
Phage protein
Phage protein
Phage lysin
10 kbp
20 kbp
30 kbp
40 kbp
50 kbp
60 kbp
70 kbp
80 kbp
Hypothetical protein
tRNA
GC Content
GC Skew+
GC Skew-
Structure protein
Functional protein
Endolysin
Terminase
Salmonella Pullorum PYW047_3
(PX021923.1)
