# Supplementary material for: WaaL–WaaP receptor cross-talk drives phage-induced collateral sensitivity to colistin in Salmonella Pullorum
Source: Vet Res. 2026 Aug 12;57:147. doi: 10.1186/s13567-026-01825-8 (PMC13471373; doi:10.1186/s13567-026-01825-8)
Supplement: Supplementary file 5 [file 13567_2026_1825_MOESM8_ESM.pdf]

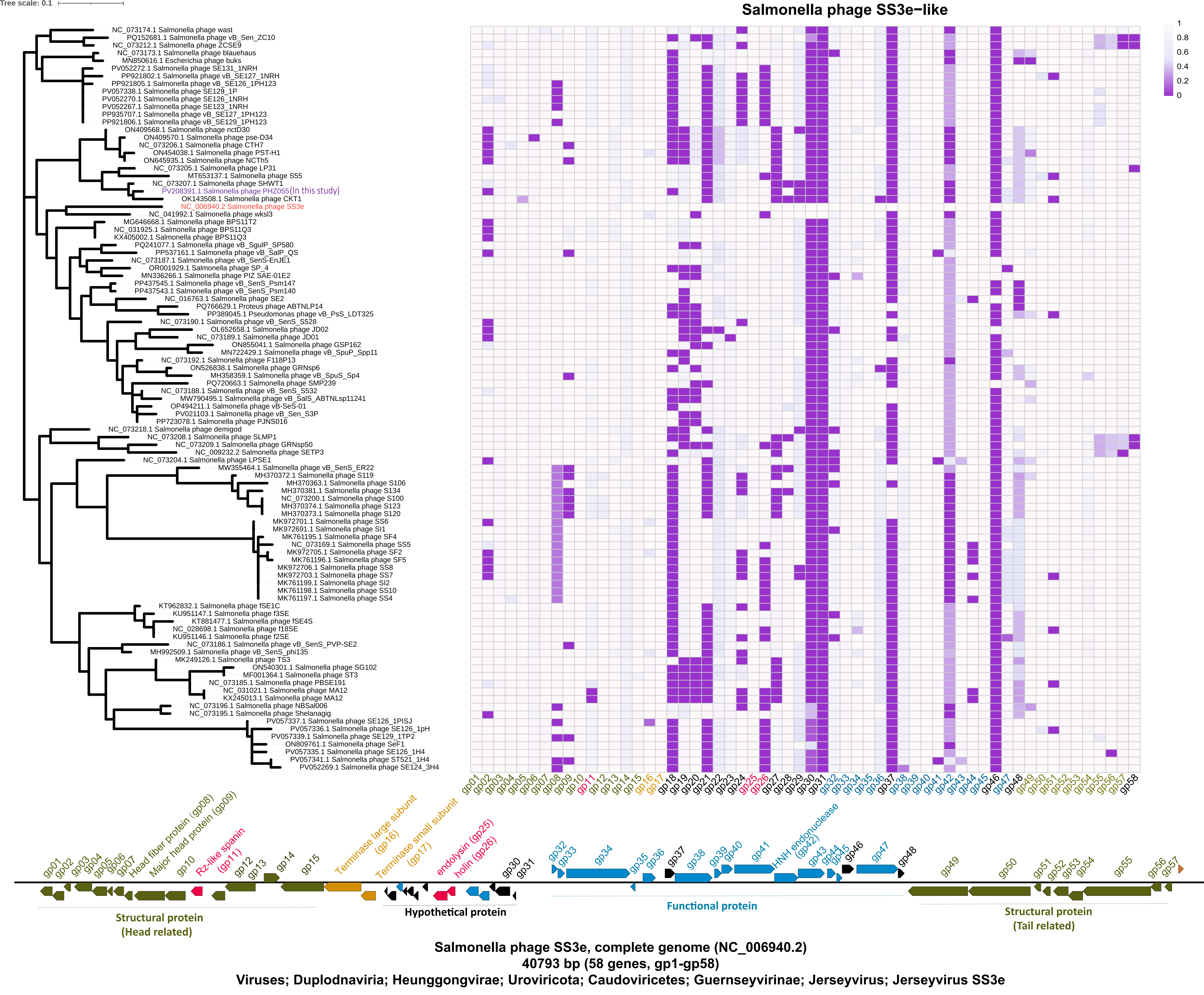

Tree scale: 0.1
Salmonella phage SS3e−like
1
0.8
0.6
0.4
0.2
0
NC_073174.1 Salmonella phage wast
PQ152681.1 Salmonella phage vB_Sen_ZC10
NC_073212.1 Salmonella phage ZCSE9
NC_073173.1 Salmonella phage blauehaus
MN850616.1 Escherichia phage buks
PV052272.1 Salmonella phage SE131_1NRH
PP921802.1 Salmonella phage vB_SE127_1NRH
PP921805.1 Salmonella phage vB_SE126_1PH123
PV057338.1 Salmonella phage SE129_1P
PV052270.1 Salmonella phage SE126_1NRH
PV052267.1 Salmonella phage SE123_1NRH
PP935707.1 Salmonella phage vB_SE127_1PH123
PP921806.1 Salmonella phage vB_SE129_1PH123
ON409568.1 Salmonella phage nctD30
ON409570.1 Salmonella phage pse-D34
NC_073206.1 Salmonella phage CTH7
ON454038.1 Salmonella phage PST-H1
ON645935.1 Salmonella phage NCTh5
NC_073205.1 Salmonella phage LP31
MT653137.1 Salmonella phage S55
NC_073207.1 Salmonella phage SHWT1
PV208391.1 Salmonella phage PHZ055(In this study)
OK143508.1 Salmonella phage CKT1
NC_006940.2 Salmonella phage SS3e
NC_041992.1 Salmonella phage wksl3
MG646668.1 Salmonella phage BPS11T2
NC_031925.1 Salmonella phage BPS11Q3
KX405002.1 Salmonella phage BPS11Q3
PQ241077.1 Salmonella phage vB_SgulP_SP580
PP537161.1 Salmonella phage vB_SalP_QS
NC_073187.1 Salmonella phage vB_SenS-EnJE1
OR001929.1 Salmonella phage SP_4
MN336266.1 Salmonella phage PIZ SAE-01E2
PP437545.1 Salmonella phage vB_SenS_Psm147
PP437543.1 Salmonella phage vB_SenS_Psm140
NC_016763.1 Salmonella phage SE2
PQ766629.1 Proteus phage ABTNLP14
PP389045.1 Pseudomonas phage vB_PsS_LDT325
NC_073190.1 Salmonella phage vB_SenS_S528
OL652658.1 Salmonella phage JD02
NC_073189.1 Salmonella phage JD01
ON855041.1 Salmonella phage GSP162
MN722429.1 Salmonella phage vB_SpuP_Spp11
NC_073192.1 Salmonella phage F118P13
ON526838.1 Salmonella phage GRNsp6
MH358359.1 Salmonella phage vB_SpuS_Sp4
PQ720663.1 Salmonella phage SMP239
NC_073188.1 Salmonella phage vB_SenS_S532
MW790495.1 Salmonella phage vB_SalS_ABTNLsp11241
OP494211.1 Salmonella phage vB-SeS-01
PV021103.1 Salmonella phage vB_Sen_S3P
PP723078.1 Salmonella phage PJNS016
NC_073218.1 Salmonella phage demigod
NC_073208.1 Salmonella phage SLMP1
NC_073209.1 Salmonella phage GRNsp50
NC_009232.2 Salmonella phage SETP3
NC_073204.1 Salmonella phage LPSE1
MW355464.1 Salmonella phage vB_SenS_ER22
MH370372.1 Salmonella phage S119
MH370363.1 Salmonella phage S106
MH370381.1 Salmonella phage S134
NC_073200.1 Salmonella phage S100
MH370374.1 Salmonella phage S123
MH370373.1 Salmonella phage S120
MK972701.1 Salmonella phage SS6
MK972691.1 Salmonella phage SI1
MK761195.1 Salmonella phage SF4
NC_073169.1 Salmonella phage SS5
MK972705.1 Salmonella phage SF2
MK761196.1 Salmonella phage SF5
MK972706.1 Salmonella phage SS8
MK972703.1 Salmonella phage SS7
MK761199.1 Salmonella phage SI2
MK761198.1 Salmonella phage SS10
MK761197.1 Salmonella phage SS4
KT962832.1 Salmonella phage fSE1C
KU951147.1 Salmonella phage f3SE
KT881477.1 Salmonella phage fSE4S
NC_028698.1 Salmonella phage f18SE
KU951146.1 Salmonella phage f2SE
NC_073186.1 Salmonella phage vB_SenS_PVP-SE2
MH992509.1 Salmonella phage vB_SenS_phi135
MK249126.1 Salmonella phage TS3
ON540301.1 Salmonella phage SG102
MF001364.1 Salmonella phage ST3
NC_073185.1 Salmonella phage PBSE191
NC_031021.1 Salmonella phage MA12
KX245013.1 Salmonella phage MA12
NC_073196.1 Salmonella phage NBSal006
NC_073195.1 Salmonella phage Shelanagig
PV057337.1 Salmonella phage SE126_1PISJ
PV057336.1 Salmonella phage SE126_1pH
PV057339.1 Salmonella phage SE129_1TP2
ON809761.1 Salmonella phage SeF1
PV057335.1 Salmonella phage SE126_1H4
PV057341.1 Salmonella phage ST521_1H4
PV052269.1 Salmonella phage SE124_3H4
gp01 gp02 gp03 gp04 gp05 gp06 gp07 gp08 gp09 gp10 gp11 gp12 gp13 gp14 gp15 gp16 gp17 gp18 gp19 gp20 gp21 gp22 gp23 gp24 gp25 gp26 gp27 gp28 gp29 gp30 gp31 gp32 gp33 gp34 gp35 gp36 gp37 gp38 gp39 gp40 gp41 gp42 gp43 gp44 gp45 gp46 gp47 gp48 gp49 gp50 gp51 gp52 gp53 gp54 gp55 gp56 gp57 gp58
gp01 gp02 gp03 gp04 gp05 gp06 gp07
Head fiber protein (gp08)
Major head protein (gp09)
gp10
Rz-like spanin (gp11)
gp12 gp13 gp14 gp15
Terminase large subunit (gp16)
Terminase small subunit (gp17)
endolysin (gp25)
holin (gp26)
gp30 gp31
gp32 gp33 gp34 gp35 gp36 gp37 gp38 gp39 gp40 gp41
HNH endonuclease (gp42)
gp43 gp44 gp45 gp46 gp47 gp48
gp49 gp50 gp51 gp52 gp53 gp54 gp55 gp56 gp57
Structural protein (Head related)
Hypothetical protein
Functional protein
Structural protein (Tail related)
Salmonella phage SS3e, complete genome (NC_006940.2)
40793 bp (58 genes, gp1-gp58)
Viruses; Duplodnaviria; Heunggongvirae; Uroviricota; Caudoviricetes; Guernseyvirinae; Jerseyvirus; Jerseyvirus SS3e
