# Supplementary material for: WaaL–WaaP receptor cross-talk drives phage-induced collateral sensitivity to colistin in Salmonella Pullorum
Source: Vet Res. 2026 Aug 12;57:147. doi: 10.1186/s13567-026-01825-8 (PMC13471373; doi:10.1186/s13567-026-01825-8)
Supplement: Supplementary file 6 [file 13567_2026_1825_MOESM9_ESM.pdf]

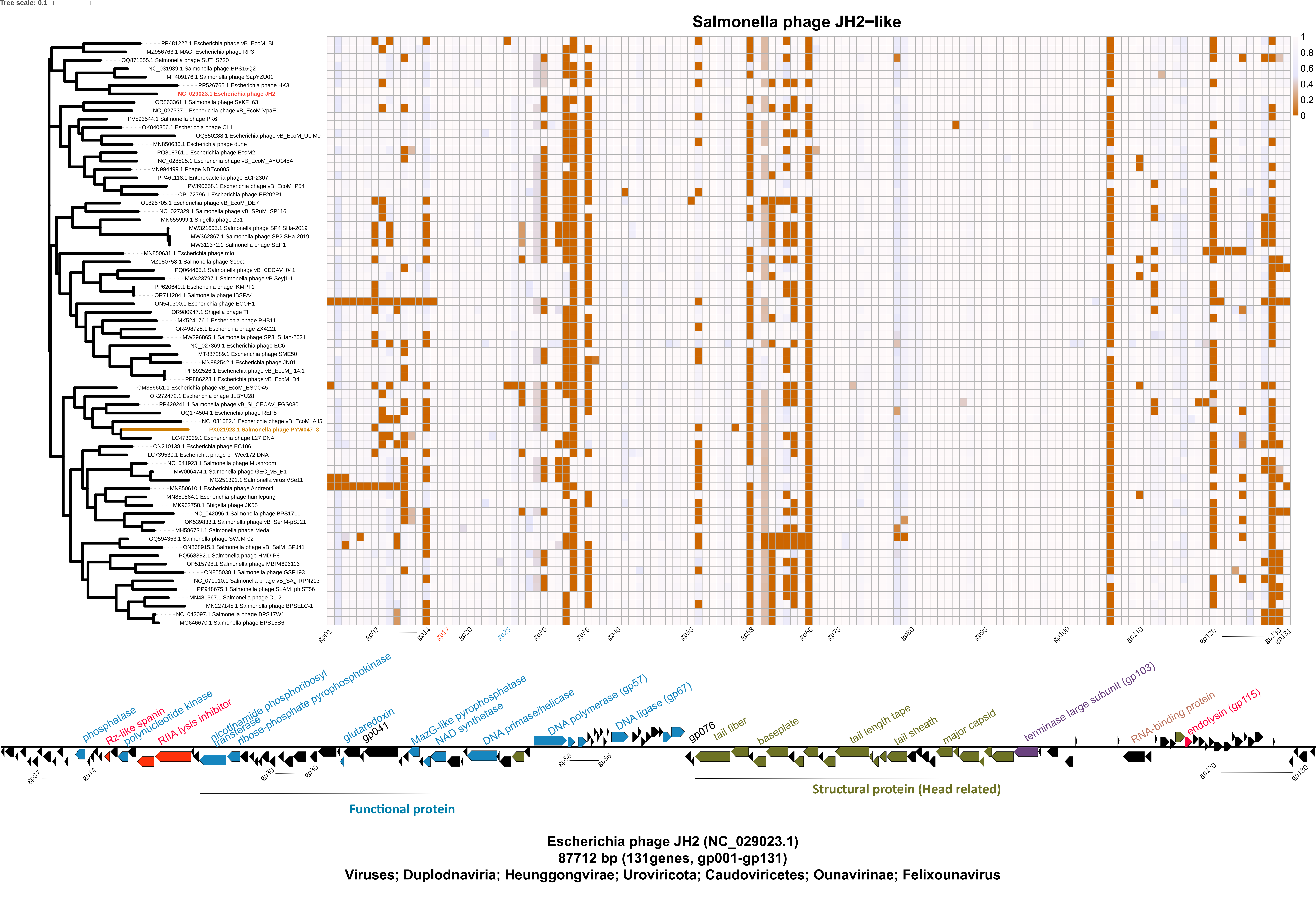
Tree scale: 0.1
Salmonella phage JH2−like
1
0.8
0.6
0.4
0.2
0
PP481222.1 Escherichia phage vB_EcoM_BL
MZ956763.1 MAG: Escherichia phage RP3
OQ871555.1 Salmonella phage SUT_S720
NC_031939.1 Salmonella phage BPS15Q2
MT409176.1 Salmonella phage SapYZU01
PP526765.1 Escherichia phage HK3
NC_029023.1 Escherichia phage JH2
OR863361.1 Salmonella phage SeKF_63
NC_027337.1 Escherichia phage vB_EcoM-VpaE1
PV593544.1 Salmonella phage PK6
OK040806.1 Escherichia phage CL1
OQ850288.1 Escherichia phage vB_EcoM_ULIM9
MN850636.1 Escherichia phage dune
PQ818761.1 Escherichia phage EcoM2
NC_028825.1 Escherichia phage vB_EcoM_AYO145A
MN994499.1 Phage NBEco005
PP461118.1 Enterobacteria phage ECP2307
PV390658.1 Escherichia phage vB_EcoM_P54
OP172796.1 Escherichia phage EF202P1
OL825705.1 Escherichia phage vB_EcoM_DE7
NC_027329.1 Salmonella phage vB_SPuM_SP116
MN655999.1 Shigella phage Z31
MW321605.1 Salmonella phage SP4 SHa-2019
MW362867.1 Salmonella phage SP2 SHa-2019
MW311372.1 Salmonella phage SEP1
MN850631.1 Escherichia phage mio
MZ150758.1 Salmonella phage S19cd
PQ064465.1 Salmonella phage vB_CECAV_041
MW423797.1 Salmonella phage vB Seyj1-1
PP620640.1 Escherichia phage fKMPT1
OR711204.1 Salmonella phage fBSPA4
ON540300.1 Escherichia phage ECOH1
OR980947.1 Shigella phage Tf
MK524176.1 Escherichia phage PHB11
OR498728.1 Escherichia phage ZX4221
MW296865.1 Salmonella phage SP3_SHan-2021
NC_027369.1 Escherichia phage EC6
MT887289.1 Escherichia phage SME50
MN882542.1 Escherichia phage JN01
PP892526.1 Escherichia phage vB_EcoM_I14.1
PP886228.1 Escherichia phage vB_EcoM_D4
OM386661.1 Escherichia phage vB_EcoM_ESCO45
OK272472.1 Escherichia phage JLBYU28
PP429241.1 Salmonella phage vB_Si_CECAV_FGS030
OQ174504.1 Escherichia phage REP5
NC_031082.1 Escherichia phage vB_EcoM_Alf5
PX021923.1 Salmonella phage PYW047_3
LC473039.1 Escherichia phage L27 DNA
ON210138.1 Escherichia phage EC106
LC739530.1 Escherichia phage phiWec172 DNA
NC_041923.1 Salmonella phage Mushroom
MW006474.1 Salmonella phage GEC_vB_B1
MG251391.1 Salmonella virus VSe11
MN850610.1 Escherichia phage Andreotti
MN850564.1 Escherichia phage humlepung
MK962758.1 Shigella phage JK55
NC_042096.1 Salmonella phage BPS17L1
OK539833.1 Salmonella phage vB_SenM-pSJ21
MH586731.1 Salmonella phage Meda
OQ594353.1 Salmonella phage SWJM-02
ON868915.1 Salmonella phage vB_SalM_SPJ41
PQ568382.1 Salmonella phage HMD-P8
OP515798.1 Salmonella phage MBP4696116
ON855038.1 Salmonella phage GSP193
NC_071010.1 Salmonella phage vB_SAg-RPN213
PP948675.1 Salmonella phage SLAM_phiST56
MN481367.1 Salmonella phage D1-2
MN227145.1 Salmonella phage BPSELC-1
NC_042097.1 Salmonella phage BPS17W1
MG646670.1 Salmonella phage BPS15S6
gp01
gp07
gp14
gp17
gp20
gp25
gp30
gp36
gp40
gp50
gp58
gp66
gp70
gp80
gp90
gp100
gp110
gp120
gp130
gp131
phosphatase
Rz-like spanin
polynucleotide kinase
RIIA lysis inhibitor
nicotinamide phosphoribosyl transferase
ribose-phosphate pyrophosphokinase
glutaredoxin
gp041
MazG-like pyrophosphatase
NAD synthetase
DNA primase/helicase
DNA polymerase (gp57)
DNA ligase (gp67)
gp076
tail fiber
baseplate
tail length tape
tail sheath
major capsid
terminase large subunit (gp103)
RNA-binding protein
endolysin (gp115)
gp07
gp14
gp30
gp36
gp58
gp66
gp120
gp130
Functional protein
Structural protein (Head related)
Escherichia phage JH2 (NC_029023.1)
87712 bp (131genes, gp001-gp131)
Viruses; Duplodnaviria; Heunggongvirae; Uroviricota; Caudoviricetes; Ounavirinae; Felixounavirus
