# Supplementary material for: WaaL–WaaP receptor cross-talk drives phage-induced collateral sensitivity to colistin in Salmonella Pullorum
Source: Vet Res. 2026 Aug 12;57:147. doi: 10.1186/s13567-026-01825-8 (PMC13471373; doi:10.1186/s13567-026-01825-8)
Supplement: Supplementary file 7 [file 13567_2026_1825_MOESM1_ESM.docx]

**Additional file 1: Bacterial strains, phages and plasmids used in this study.**

| **Bacterial strains/phages/plasmids** | | **Description** | **Source** |
| --- | --- | --- | --- |
| *Salmonella* pullorum | HZ055 WT | Wild type *Salmonella* pullorum strain | This study |
|  | YW047 WT | Wild type *Salmonella* pullorum strain | This study |
|  | HZ055-R P055 | Phage-resistant bacteria in vitro | This study |
|  | HZ055-R 2P | Phage-resistant bacteria in vitro | This study |
|  | YW047-R P047-3 | Phage-resistant bacteria in vitro | This study |
|  | HZ055△*YehA* | Knock out *YehA* in·HZ055 background | This study |
|  | HZ055△*WaaL* | Knock out *WaaL* in·HZ055 background | This study |
|  | YW047△*rfaJ* | Knock out *rfaJ* in·YW047 background | This study |
|  | YW047△WaaP | Knock out *WaaP* in·YW047 background | This study |
|  | DH5α-λpir | Transformation competent state harboring suicide plasmid pRE112 | Lab store |
|  | X7213 | Conjugative model strain harboring suicide plasmid pRE112 | Lab store |
| Phages | PHZ055 | N4-like phage that·can lyse·*Salmonella* pullorum strain HZ055 | This study |
|  | PYW047-3 | N4-like phage that·can lyse·*Salmonella* pullorum strain YW047 | This study |
| Plasmids | pRE112 | Cm^R^ a suicide plasmid used to link the forward and backward segments of the target gene | Lab store |
|  | pRE112- *YehA* | Cm^R^ plasmid for ligating the forward and backward·segments of HZ055 *YehA* gene | This study |
|  | pRE112- *WaaL* | Cm^R^ plasmid for ligating the forward and backward·segments of HZ055 *WaaL* gene | This study |
|  | pRE112- *rfaJ* | Cm^R^ plasmid for ligating the forward and backward·segments of YW047 *rfaJ* gene | This study |
|  | pRE112- *WaaP* | Cm^R^ plasmid for ligating the forward and backward·segments of YW047 WaaP gene | This study |
|  | pBAD-44 | Kan^R^ arabinose-inducible expression vector used for complementation assays. | Lab store |
|  | pBAD -44- *YehA* | Kan^R^ complementation plasmid carrying the YehA gene of HZ055 under pBAD control. | This study |
|  | pBAD -44- *WaaL* | Kan^R^ complementation plasmid carrying the *WaaL* gene of HZ055 under pBAD control | This study |
|  | pBAD -44- *rfaJ* | Kan^R^ complementation plasmid carrying the *rfaJ* gene of HZ055 under pBAD control | This study |
|  | pBAD -44- *WaaP* | Kan^R^ complementation plasmid carrying the *WaaP* gene of HZ055 under pBAD control | This study |

Cm^R^ indicate Chloramphenicol resistance; Kan^R^ indicate Kanamycin resistance.
