# Supplementary material for: WaaL–WaaP receptor cross-talk drives phage-induced collateral sensitivity to colistin in Salmonella Pullorum
Source: Vet Res. 2026 Aug 12;57:147. doi: 10.1186/s13567-026-01825-8 (PMC13471373; doi:10.1186/s13567-026-01825-8)
Supplement: Supplementary file 8 [file 13567_2026_1825_MOESM2_ESM.docx]

**Additional file 2: All URL links originally appearing in the main text.**

| **Tool / Resource Name** | **Uniform Resource Locator (URL)** |
| --- | --- |
| **ABRicate v1.0.1** | <https://github.com/tseemann/abricate> |
| **RAST online platform** | <https://rast.nmpdr.org/> |
| **CGView Server** | <https://www.bioinformatics.org/cgview/> |
| **ICTV website** | <https://ictv.global/> |
