# Supplementary material for: WaaL–WaaP receptor cross-talk drives phage-induced collateral sensitivity to colistin in Salmonella Pullorum
Source: Vet Res. 2026 Aug 12;57:147. doi: 10.1186/s13567-026-01825-8 (PMC13471373; doi:10.1186/s13567-026-01825-8)
Supplement: Supplementary file 9 [file 13567_2026_1825_MOESM3_ESM.docx]

**Additional file 3:: Nucleotide sequences of primers used in this study.**

| **Target** | | **Primer Sequence (listed5’to3’)** | **Target Size (bp)** |
| --- | --- | --- | --- |
| **PCR for construction of deletion mutants** | pRE112- *YehA-*1F | tgaactgcatgaattcccgggGCCTTAATGATACTATCGCGATTATT | 500 |
|  | pRE112- *YehA-*1R | taccgcataaatGGCGTCACCTAAAGATTACCGA |  |
|  | pRE112- *YehA-*2F | gtgacgccATTTATGCGGTACGCACTCTTTATA | 500 |
|  | pRE112- *YehA-*2R | cgatcccaagcttcttctagaCGGATATGTTGCTGCACGACG |  |
|  | *YehA-*3F | gtcggcggcagttacag | 2227 |
|  | *YehA-*3R | gtctgcgccagacaatg |  |
|  | pRE112- *WaaL -*1F | tgaactgcatgaattcccgggTCGCAAAACATTTTCTGCATTG | 500 |
|  | pRE112- *WaaL -*1R | gcgcgttttttCTTTTCTCCACAATAGGTTTGGG |  |
|  | pRE112- *WaaL -*2F | ggagaaaagAAAAAACGCGCTGATACTTATTACG | 500 |
|  | pRE112- *WaaL -*2R | cgatcccaagcttcttctagaCAAGCCGCAAGGGTGAAA |  |
|  | *WaaL -*3F | atttcgcctgataaaggcat | 2415 |
|  | *WaaL -*3R | caccatatcgcaaagcaac |  |
|  | pRE112- *rfaJ -*1F | tgaactgcatgaattcccgggTCTGGCTATTTTAATGCCGGTT | 500 |
|  | pRE112- *rfaJ -*1R | taggcaggggaaTACATCACCTATGGGTTTTATTCAAGA |  |
|  | pRE112- *rfaJ -*2F | gtgatgtaTTCCCCTGCCTAAATCTCTATTATG | 500 |
|  | pRE112- *rfaJ -*2R | cgatcccaagcttcttctagaCGCCCGAAACCATACCATG |  |
|  | *rfaJ -*3F | atacgatcctttgcttttcgt | 2214 |
|  | *rfaJ -*3R | tcttaattttgctgaaaatgagattgc |  |
|  | pRE112- *WaaP -*1F | tgaactgcatgaattcccgggTACCCGTAAAGGTGTGGATCG | 500 |
|  | pRE112- *WaaP -*1R | tattataaCTAAATCACCTGTAATAATATCCGCG |  |
|  | pRE112- *WaaP -*2F | caggtgatttagTTATAATAATTATGCACTCAGTGAGTAATTTATG | 500 |
|  | pRE112- *WaaP -*2R | cgatcccaagcttcttctagaTAATCTCTTTTAGCCATGCTTTATCC |  |
|  | *WaaP -*3F | gaaaatatagccaacagatcccaa | 2026 |
|  | *WaaP -*3R | acatgagatgatatcaatacatatcaca |  |
|  | pRE112-CHL-id-F | tttagccccgacatagcc | 1200 |
|  | pRE112-CHL-id-R | atgtcggcagaatgcttaatg |  |
| **PCR for construction of complemented strains** | pBAD-*WaaL-*F | ttgggctagcaggaggaattcATGCTAACCACATCATTAACGTTAAA | 1257 |
|  | pBAD-*WaaL-*R | tccgccaaaacagccaagcttTTATCTATTTCTTAGCGCCAACAGA |  |
|  | pBAD-*WaaP-*F | ttgggctagcaggaggaattcATGGTTGAGCTGAAAGCGCC | 840 |
|  | pBAD-*WaaP-*R | tccgccaaaacagccaagcttTTACAGATTTTTTCTTATTGTTCTTTCTTT |  |
|  | pBAD-44-id-F | aaaagtccacattgattatttgcac | 1515/1098 |
|  | pBAD-44-id-R | gcgtttcacttctgagttcg |  |
| **qpcr for gene expression level** | *EptA*-QF | AGTGCTACGATGAAGTGTTA | 92 |
|  | *EptA*-QR | GCTGCCGATGGTATGTAA |  |
|  | BasR-QF | GAAGGCTATGCGTGTGAT | 67 |
|  | BasR-QR | AATGACCAGACTCCAGACT |  |
|  | pmrB-QF | GTTACTGCGTATGCTGCT | 84 |
|  | pmrB-QR | GCTAATGTGGATAGTGATATGG |  |
|  | pmrR-QF | CCTGTGTAGTGTGCTGATT | 50 |
|  | pmrR-QR | TCGTGACCCACACATAGA |  |
|  | pmrD-QF | TTCTGGTGCTGTGCGATA | 73 |
|  | pmrD-QR | GGGACTCAGTAATATCATGGAA |  |
|  | pmrK-QF | GAGACGATATTATGCTCTACG | 53 |
|  | pmrK-QR | GCTAAGACCATACCTGAGTT |  |
|  | pmrG-QF | TGGAGTGGTAACGGTCTA | 85 |
|  | pmrG-QR | GGCGGAATAACACGACTA |  |

F indicates forward primer and R indicates reverse prime
