# Supplementary material for: WaaL–WaaP receptor cross-talk drives phage-induced collateral sensitivity to colistin in Salmonella Pullorum
Source: Vet Res. 2026 Aug 12;57:147. doi: 10.1186/s13567-026-01825-8 (PMC13471373; doi:10.1186/s13567-026-01825-8)
Supplement: Supplementary file 10 [file 13567_2026_1825_MOESM4_ESM.docx]

**Additional file 4: Lytic Spectrum of PHZ055 and PYW047-3**

| **Strain** | **PHZ055** | **PYW047-3** |
| --- | --- | --- |
| **ATCC 9120** | +++++ | +++ |
| **ATCC 14028** | - | +++ |
| **ATCC 13076** | +++++ | - |
| **HZ055** | +++++ | + |
| **055R** | - | +++ |
| **YW047-3** | - | +++ |
| **047R** | - | - |
| **R2P** | - | - |
| **30** | +++++ | +++ |
| **24137** | +++++ | +++ |
| **342** | +++++ | +++ |
| **322** | - | +++ |
| **23300** | - | ++++ |

+++++: Strong lytic activity, clear and well-defined plaques at high dilution

+++: Moderate lytic activity

+: Weak lytic activity

-: No lysis
